# Distribution-dependent representations in auditory category learning and generalization

**DOI:** 10.3389/fpsyg.2023.1132570

**Published:** 2023-09-27

**Authors:** Zhenzhong Gan, Lurong Zheng, Suiping Wang, Gangyi Feng

**Affiliations:** ^1^Philosophy and Social Science Laboratory of Reading and Development in Children and Adolescents (South China Normal University), Ministry of Education, Guangzhou, Guangdong, China; ^2^Guangdong Provincial Key Laboratory of Mental Health and Cognitive Science, South China Normal University, Guangzhou, Guangdong, China; ^3^School of Psychology, South China Normal University, Guangzhou, Guangdong, China; ^4^Department of Linguistics and Modern Languages, The Chinese University of Hong Kong, Shatin, Hong Kong SAR, China; ^5^Brain and Mind Institute, The Chinese University of Hong Kong, Shatin, Hong Kong SAR, China

**Keywords:** auditory category learning, generalization, representation nature, dual learning system, information integration, rule-based learning

## Abstract

A fundamental objective in Auditory Sciences is to understand how people learn to generalize auditory category knowledge in new situations. How we generalize to novel scenarios speaks to the nature of acquired category representations and generalization mechanisms in handling perceptual variabilities and novelty. The dual learning system (DLS) framework proposes that auditory category learning involves an explicit, hypothesis-testing learning system, which is optimal for learning rule-based (RB) categories, and an implicit, procedural-based learning system, which is optimal for learning categories requiring pre-decisional information integration (II) across acoustic dimensions. Although DLS describes distinct mechanisms of two types of category learning, it is yet clear the nature of acquired representations and how we transfer them to new contexts. Here, we conducted three experiments to examine differences between II and RB category representations by examining what acoustic and perceptual novelties and variabilities affect learners’ generalization success. Learners can successfully categorize different sets of untrained sounds after only eight blocks of training for both II and RB categories. The category structures and novel contexts differentially modulated the generalization success. The II learners significantly decreased generalization performances when categorizing new items derived from an untrained perceptual area and in a context with more distributed samples. In contrast, RB learners’ generalizations are resistant to changes in perceptual regions but are sensitive to changes in sound dispersity. Representational similarity modeling revealed that the generalization in the more dispersed sampling context was accomplished differently by II and RB learners. II learners increased representations of perceptual similarity and decision distance to compensate for the decreased transfer of category representations, whereas the RB learners used a more computational cost strategy by default, computing the decision-bound distance to guide generalization decisions. These results suggest that distinct representations emerged after learning the two types of category structures and using different computations and flexible mechanisms in resolving generalization challenges when facing novel perceptual variability in new contexts. These findings provide new evidence for dissociated representations of auditory categories and reveal novel generalization mechanisms in resolving variabilities to maintain perceptual constancy.

## Introduction

Category learning ability allows us to learn to group items based on shared features or common regularities across individual examples. The grouping knowledge acquired during learning is considered abstract category representations guiding us to categorize items in new scenarios (Kruschke, 1996; Smith and Minda, 1998, 2002; Love et al., 2004; Wills et al., 2006). Successful generalization of learned category knowledge requires coping with various novel variabilities of within- and between-category exemplars. Failure to do so would affect critical decisions in our lives and even survival (e.g., under-generalization: missing detecting early signatures of serious diseases, or over-generalization: categorizing poison mushrooms as eatable). Therefore, category learning requires forming mental representations to support an appropriate level of generalization. However, we are not yet fully understand the nature of these category representations and the differences in representational characteristics when learning categories with different structures.

Category representations are formed in the process of interacting with category members, where the structure and distributional patterns of the category members affect the learning process and the nature of acquired category representations (Ashby and Maddox, 2005, 2011; Chandrasekaran et al., 2014a,b). Previous behavioral and neuroimaging studies suggest that at least two distinct learning systems are involved in category learning across visual (e.g., Ashby and Maddox, 2005, 2011; Smith and Grossman, 2008) and auditory modalities (Chandrasekaran et al., 2014a,b; Feng et al., 2021b). An explicit, hypothesis-testing learning system is considered to be optimal for learning rule-based (RB) categories, where those categories can be categorized based on verbalized rules. In contrast, an implicit, procedural-learning-based system is hypothesized to be optimal for learning category structures requiring pre-decisional information integration (II) across multiple dimensions (Ashby and Maddox, 2005; Ashby and Valentin, 2017).

Previous studies have demonstrated various factors that differentially affect the learning of II and RB category structures. For example, feedback types (instant vs. delayed; with vs. without feedback) affect learning II categories more than RB’s (Maddox et al., 2003; Maddox and Ing, 2005); category-response mapping is more critical and specific for II category learning than that of RB (Ashby et al., 2003; Maddox et al., 2004b, 2007, 2010). In contrast, RB category learning is more affected by executive-function-related factors, e.g., concurrent working memory task (Waldron and Ashby, 2001; Filoteo et al., 2010), stress (Ell et al., 2011), feedback processing interference (Maddox et al., 2004a), number of categories (i.e., limitation of working memory) (Maddox et al., 2004c). These findings support the dual learning system (DLS) framework that distinct neural and cognitive systems are involved in learning II and RB categories.

Different category learning tasks recruiting distinct learning systems provides a helpful framework to reason that distinct natures of category representations emerged after learning (due to the engagement of different learning processes and distinct mechanisms support learners to generalize acquired representations to categorize new items that are different from training samples in various levels of similarity). However, these predictions have not been systematically tested to date, as we know, and there is no detailed description of the representation nature and generalization mechanisms underlying the DLS. In the present study, we aim to test these predictions and reveal the abstraction levels of category representations of II and RB categories by modeling learners’ categorization responses with representational similarity models and examining how well learners generalize the acquired category knowledge to novel contexts.

Generalization and categorization require accessing our learned category knowledge to guide the judgment of within- and between-category members. Types and degrees of sampling variabilities affect how well we generalize the learned category knowledge. To resolve generalization challenges imposed by stimulus novelties and variabilities, we must form category representations abstracted from individual exemplars-specific features at appropriate levels (Raviv et al., 2022). The abstract form of category representations was previously considered prototypes or rules. Prototype-based representation theories propose that category membership is determined by the distance or similarity between members and a category prototype abstracted from individual training samples (Posner and Keele, 1968; Reed, 1972; Rosch, 1973; Homa et al., 1981; Smith and Minda, 2000). Rule or regularity derived from the hypothesis-testing-based learning process is another abstract form of category representations. A rule may refer to a dimensional boundary (e.g., Category A consists of larger items, while Category B consists of smaller ones; a decision is based on the dimension of size) or complex combinations of features (simultaneous presentation of features a and b belongs to Category A, while the presentation of features a and c belongs to Category B) (Erickson and Kruschke, 1998; for other instantiations of RB models, please see Ashby and Maddox, 1992; Maddox and Ashby, 1993). RB and prototype-based hypotheses would predict that surface variations in a new context are less likely to affect the generalization of these abstract representations.

Successful generalization may also depend on exemplar-specific representations acquired from the physical and perceptual properties of training samples (Erickson and Kruschke, 1998, 2002). Exemplar-based representation theories assume that categorizing new stimuli are based on the similarities between new items and previously-experienced individual exemplars in a psychological space (Medin and Schaffer, 1978; Estes, 1986; Nosofsky, 1986, 2011). The emergence of exemplar- or feature-specific representations has been considered dominant in learning categories with a II structure. For example, in the visual domains, II category learners performed significantly less accurately when categorizing new items sampled from untrained locations in the stimulus space (Casale et al., 2012). Similarly, changes in stimulus dimensions significantly affected generalization performances even though the dimensions are irrelevant to categorization (Filoteo et al., 2010; Grimm and Maddox, 2013). Rosedahl et al. (2018) reported a more striking finding: learners exposed to items presented in one visual field failed to categorize the same items presented in another, suggesting visual-field-dependent representations emerged after learning visual II categories. However, other studies revealed a different pattern that II category learners can generalize learned representations to categorize items from untrained perceptual locations in the visual domain (Nosofsky et al., 2005; Seger et al., 2015). These mixed findings suggest that the abstractness of II category representations are required further investigation.

**Figure 1:**
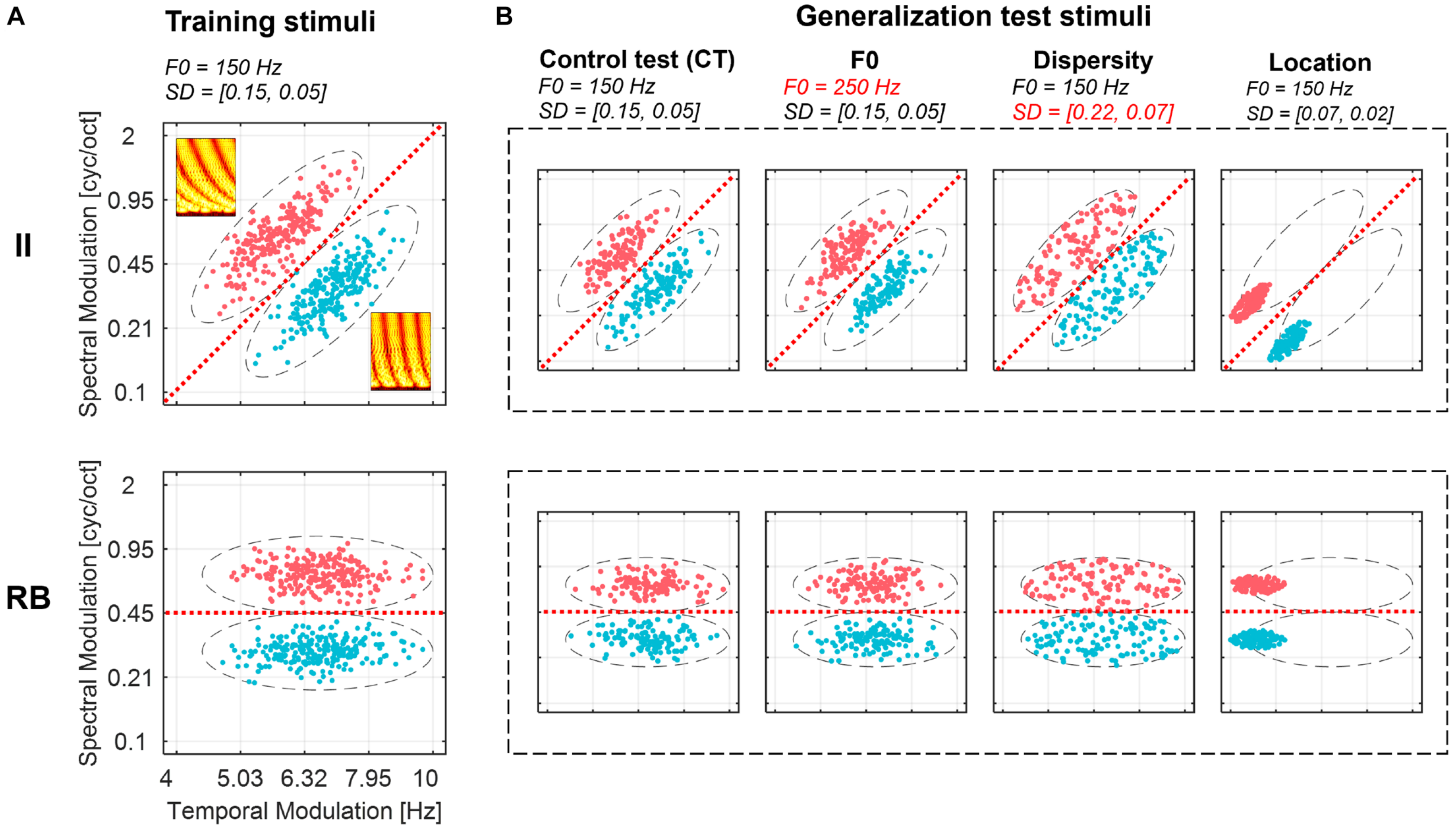
Ripple sounds of II and RB category structures used for training **(A)** and generalization tests **(B)**. Two categories of sounds are plotted in different colors (Category 1: red, Category 2: blue) in a two-dimensional (i.e., spectral and temporal modulation dimensions) space. The red dashed lines represent the optimal boundaries separating the two categories. Dashed ellipses represent the coverage regions of the stimuli. Sampling parameters (i.e., F0 and dispersity [SD]) are listed at the top of each graph. Two spectrograms of the samples were plotted in the subgraph A. F0, fundamental frequency; SD, standard deviation of each within-category sampling distribution ([SD_long_, SD_short_]).

Here we designed novel generalization paradigms to examine the nature and abstractness of representations that emerged after learning auditory categories with II and RB structures. We generated well-controlled non-speech auditory category structures with the same perceptual dimensions (i.e., spectral and temporal modulations). These dimensions are fundamentals of complex acoustic signals like music and speech. We sampled sounds from two-category II and RB structures in the two-dimensional space, where the RB structure prefers categorization using an explicit criterion on the spectral-modulation dimension (high vs. low), and the II structure requires learning to integrate information from the two dimensions simultaneously (a diagonal category bound). Importantly, we devised four generalization tests (see Figure 1 for a graphical illustration) by sampling new sounds that were different from the training samples in different degrees to probe the abstractness of the learned category knowledge. The four tests consist of a baseline generalization control test (CT) without inducing new variations, but the items were not exposed in the training sessions; a “fundamental frequency (F0)” test where a change in a new acoustic dimension (i.e., F0) irrelevant to the categorization was induced; a “Dispersity” test where the dispersity of the generalization sounds increased compared to the training samples while the sampling range in the perceptual space remains unchanged; and a “Location” test where the generalization sounds were sampled from an untrained region of the perceptual space. We consider CT as the baseline test of generalization because the sounds in this test were generated from the same sampling distribution as the training samples. Thus, any differences in performance between CT and the other three tests are presumable due to the differences between training and generalization samples in sound properties. Successful generalization to the four tests requires learners to form and transfer representations with different abstraction levels, ranging from generalizing category knowledge across items with surface acoustic differences (in the F0 test) to items with different distributional patterns (i.e., in the Dispersity and Location tests).

According to the DLS framework, learning RB categories is dominated by an explicit learning system associated with working memory and executive hypothesis-testing functions (Chandrasekaran et al., 2014a,b; Feng et al., 2021b). Thus, DLS predicts that RB category representations are abstracted from individual training samples and their associated acoustic and perceptual features. RB learners can generalize the learned category knowledge to untrained items with different types of novel variabilities. If this is the case, we would predict that RB learners perform equally well across the four generalization tests. In contrast, II auditory category learning is hypothesized to be dominated by an implicit learning system that maps sounds onto categories *via* corticostriatal systems (Chandrasekaran et al., 2014a,b; Feng et al., 2021b). This implicit sound-to-category mapping learning is not consciously penetrable and requires accessing and integrating information from multiple dimensions (Ashby et al., 2003; Maddox et al., 2007; Smith et al., 2015; Rosedahl et al., 2018). Therefore, the DLS predicts that II category knowledge tightly pertains to sample-specific features and the relative weighting of the features in the perceptual space. If this hypothesis is correct, we would predict that II learners will decrease generalization performances in F0, Dispersity, and Location tests compared to the baseline CT test. The degree of decrease in generalization performance depends on the abstractness of the acquired representations. If the emergent II representations are strictly specific to exemplar features, any surface-level changes will affect generalization performance. However, if the representations are not strictly exemplar-specific, II learners could resist certain degrees of acoustic and perceptual changes. Thus, the generalization performance will be as a function of the four tests (e.g., baseline > F0 > Dispersity > Location).

We conducted three auditory category learning experiments to test this representational DLS model and the above predictions, focusing on the nature of II and RB category representations and generalization mechanisms. Across three experiments, we also examined whether categorization-based training (Exp. 2.1) and providing feedback (Exp. 2.2) could facilitate category learning and generalization and alter the nature of emergent representations compared to observational training (Exp. 1). Numerous studies showed that category training procedures modulate learning outcomes (e.g., Ashby et al., 1999, 2002; Goudbeek et al., 2008, 2009; Levering and Kurtz, 2015). Compared to observational training, categorization training could facilitate category learning by learning to attend to discriminative features between categories while ignoring within-category item variabilities (Hsu and Griffiths, 2010; Levering and Kurtz, 2015). In addition, providing immediate feedback was considered a prerequisite for learning II categories (Maddox et al., 2003; Maddox and Ing, 2005). Specifically, feedback facilitates performances in learning auditory and speech categories (Goudbeek et al., 2009). Participants were constrained to use a sub-optimal strategy to learn categories without feedback (Ashby et al., 1999). We examined to which extent the two factors augment the differences between II and RB category representations. In addition to the categorization accuracies averaged across trials, we modeled trial-by-trial categorization responses with representational similarity analysis (RSA) (Kriegeskorte et al., 2008) for each generalization test to reveal the nature of emergent representations of each category structure. With pre-defined representational models, RSA enables us to reveal to what extent different stimulus-related information besides category knowledge is acquired during training and utilized in generalization when facing different stimulus changes.

## Materials and methods

### Participants

A total of 104 adults (88 females, age range = 18–27 years) were recruited from South China Normal University communities for three auditory-category learning and generalization experiments. For each experiment, participants were assigned to one of the training groups according to the category structures (i.e., II or RB). Two participants in Exp. 1 were removed from further analyses because they misunderstood the task instruction (i.e., performed categorization). There were 35 participants left (II group: mean age = 21.12, *SD* = 2.74, *N* = 17; RB group: mean age = 19.39, *SD* = 1.69, *N* = 18). Two participants in Experiment 2 were removed because they missed more than one-third of test trials in at least three generalization tests. There are 32 participants in Exp. 2.1 (II group: mean age = 19.50, *SD* = 1.37, *N* = 16; RB group: mean age = 20.31, *SD* = 1.58, *N* = 16), and 33 participants in Exp. 2.2 (II group: mean age = 19.75, *SD* = 1.98, *N* = 16; RB group: mean age = 20, *SD* = 2.15, *N* = 17). All participants reported normal hearing and had normal or corrected-to-normal vision. No participant reported a history of neurological disorders or reading disabilities. All materials and protocols were approved by the ethics review board of the School of Psychology at South China Normal University and the Joint Chinese University of Hong Kong – New Territories East Cluster Clinical Research Ethics Committee. Written informed consent was obtained before the experiment.

### Stimulus construction

The ripple sounds were generated by modulating a broadband noise stimulus along spectral and temporal dimensions. The frequency band of this broadband noise ranges from 150 Hz (i.e., the fundamental frequency, F0) to its fifth octaves (i.e., 4.8 kHz). The spectral modulation is an energy modulation along the frequency of the sounds with a sinusoidal envelope, ranging from 0.1 to 2 cycle/octave. The larger values of the cycle/octave indicate more energy fluctuations in the frequency dimension (Figure 1; see detailed graphical illustration of spectral modulation in Supplementary Figure S1). The temporal modulation is a frequency modulation of the phase change of the sinusoidal envelope, with a range from 4 to 10 Hz. This means that the phase of the sinusoidal envelope changes over time at a rate that varies between 4 and 10 cycles per second (Hz). The larger temporal modulation values (e.g., 10 Hz) indicate a faster change of a frequency component in time (see detailed graphical illustration of temporal modulation in Supplementary Figure S1). We selected these modulation ranges since they are strongly represented in the human auditory cortex (Schönwiesner and Zatorre, 2009) and reflect the complexity of natural spectro-temporal variations in speech (Elliott and Theunissen, 2009). Meanwhile, previous studies show that the spectral and temporal modulation dimensions are independent of each other at multiple levels of processing (Depireux et al., 2001; Langers et al., 2003; Schönwiesner and Zatorre, 2009). The change in F0 is also independent of the changes in spectral and temporal modulations. All sounds were synthesized with in-house scripts with MATLAB (Mathworks, Natick, MA, USA), and the RMS amplitude was normalized to 80 dB.

Two sets of ripple sounds were generated for category training based on predefined two-categories II and RB structures, respectively. We created two bivariate distributions in a normalized two-dimensional stimulus space along the positive diagonal to sample sounds of the II categories. This normalized space ranges from 0 to 1 along spectral and temporal modulation dimensions. The centers of two II categories are in the locations of [temp. = 0.39, spec. = 0.61] and [temp. = 0.61, spec. = 0.39]. The standard deviation (SD) is 0.15 for the longer axis and 0.05 for the shorter axis with an orthogonal design. The optimal category bound is along the positive diagonal (Figure 1A upper panel, red dashed lines). To estimate the relationship between participants’ decision bound and their categorization accuracy, we conducted a simulation by varying the angles of the bound for categorization. We found that if a participant uses a vertical or horizontal line as the category bound to categorize, the maximal performance the participants can achieve is 83%. Achieving higher performances require procedure-based learning and a shift in decision strategy.

We randomly sampled 480 ripple sounds for the II category structure. Values along each dimension were logarithmically mapped onto temporal and spectral modulation dimensions. The RB category sounds were generated by rotating the II samples by 45° anticlockwise, and the centers of the two categories were shifted to [temp. = 0.55, spec. = 0.35] and [temp. = 0.55, spec. = 0.65]. The optimal category bound for the RB sounds perpendicular to the spectral modulation dimension at 0.45 cyc/oct (Figure 1A lower panel, red dashed lines).

To uncover the representation nature of auditory II and RB categories, we examined the extent to which the learners generalized the learned category-related knowledge to four different sets of generalization sounds that deviated from the training samples in acoustic and distributional properties (see Figure 1B for a graphical illustration). In the control test (CT), we used new sounds that were sampled from the same distribution as the training samples, but were not exposed during the training process. The CT acts as a baseline to measure generalization effects of other tests. If there is a decrease in generalization performance in subsequent tests compared to the CT, it could be due to changes in acoustic or distributional factors. In the “fundamental frequency (F0)” test, we introduced a new acoustic dimension (i.e., F0) that was irrelevant to the categorization. The F0 of these new sounds was changed from 150 Hz (a default setting for training and other test sounds) to 250 Hz. This manipulation is similar to changing from a male to a female speaker to produce sounds. In the Dispersity test, we increased the sampling dispersity (i.e., more sampling variations for each category than the training sounds). The sampling dispersity increased in both the long (parallel to the optimal boundary) and short (orthogonal to the boundary) axes (from [SD_long_ = 0.15, SD_short_ = 0.05] to [SD_long_ = 0.22, SD_short_ = 0.07]) compared to the training set, while the coverage areas in the stimulus space (i.e., Figure 1, dashed ellipse) remain unchanged. Increasing the dispersity of sounds results in an equal number of sounds that are closer and farther from the optimal categorization bound compared to CT. Thus, the difference in categorization performance between Dispersity and CT conditions can be attributed to the difference in sampling dispersity. In the Location test, we sampled novel sounds where their center was outside the coverage range of the training samples. The category distributions were moved away along the optimal bound to the left edge of the training samples, resulting in new centers of the two categories (i.e., the coordinate of the centers; II category 1: [temp. = 0.11, spec. = 0.32], category 2: [temp. = 0.32, spec. = 0.11]; RB category 1: [temp. = 0.15, spec. = 0.35], category 2: [temp. = 0.15, spec. = 0.65]). The new centers were 2.67 SDs away from the centers of the training samples. Note that changing the location of the center also links with decreased sampling dispersity in this test because preserving sampling dispersity would introduce sounds in the auditory locations that are not sensitively perceived by human auditory systems. Therefore, we reduce the dispersity of the sounds in the Location test. Nevertheless, we assume training with more dispersed samples and testing on less dispersed samples would not hurt the categorization performance. Thus, any decrease in categorization performance in the Location condition is due to the change in perceptual location. These four types of manipulation were applied equally to II and RB categories to generate four generalization tests (Figure 1B). We generated 240 sounds for each generalization test.

### Training and generalization procedures

**Figure 2:**
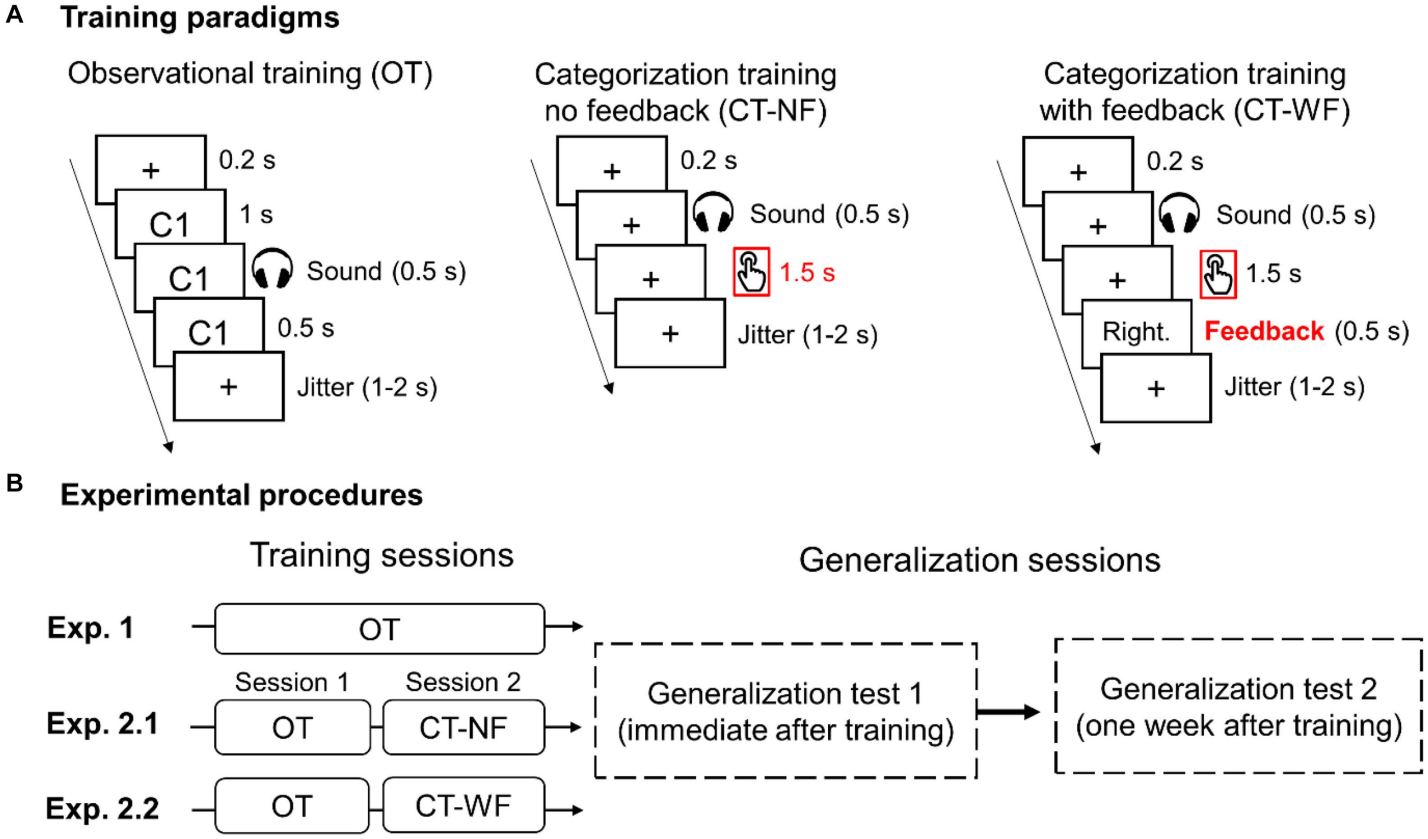
Illustration of training paradigms **(A)** and experimental procedures **(B)** used to train participants to learn auditory categories in the three experiments. **(A)** Three category training paradigms. The time windows for categorization response and visual feedback are highlighted in red. **(B)** Training and generalization procedures for the three experiments.

We conducted three experiments by manipulating the training procedure to examine whether categorization and providing feedback could facilitate category learning and generalization. In Exp. 1, participants were asked to learn to associate category labels with sounds *via* observational training (OT) procedure (Figure 2A, left panel). For each training trial, a fixation cross was presented for 200 ms, followed by a category label (i.e., “类别 1” or “类别 2”) displayed on the screen. One second after the category label appeared, a sound was presented for 500 ms. Participants were instructed to learn the associations between sounds and category labels without making any categorization response. After each trial, a jittered inter-trial interval (randomly sampling between 1 and 2 s) was added to minimize the effects of temporal prediction. Each participant completed eight blocks of OT. Each training block consists of 60 trials.

Participants were asked to complete the four generalization tests twice, one immediately following training and another 1 week after (Figure 2B). The CT was always performed first to minimize the influence from other tests. The order of the other three tests (i.e., F0, Dispersity, and Location) was counterbalanced across participants. Each generalization test consists of two blocks (60 trials/block). The training and testing were conducted in a sound-attenuated booth using E-Prime software (Psychology Software Tools, Inc., Sharpsburg, PA, USA). The sounds were presented over headphones (Sennheiser HD280 pro) at a comfortable listening level.

In Exp. 2.1, participants first completed four blocks of OT (session 1) and performed another four blocks of categorization training with no feedback (CT-NF) in session 2 (Figure 2B). The OT procedure was the same as Exp. 1. In the CT-NF blocks, for each trial, a fixation cross was presented for 200 ms, followed by a ripple sound for 500 ms. Participants were instructed to categorize sounds into one of the two categories (Figure 2A, middle panel). In Exp. 2.2, the same training paradigm was used as the Exp. 2.1 except for providing corrective feedback for each categorization response (i.e., CT-WF, see Figure 2A right panel) in the training session 2. Exp. 2.2 consisted of four blocks of OT and then four blocks of CT-WF (Figure 2B). Visual feedback (i.e., “正确” “Right” or “错误” “Wrong”) was displayed for 500 ms after each response. If participants failed to respond within 2 s following the sound onset, cautionary feedback was presented (i.e., “No response”). A jittered inter-trial interval was added after each trial. The generalization test is a categorization task without providing feedback, the same procedure as the CT-NF. The generalization test procedure was the same across the three experiments.

### Data analysis

#### Representation modeling

Although categorization accuracy provides an overall performance for each generalization test, it does not tell us how participants’ sound-by-sound variabilities in response patterns and category confusions associate with category knowledge acquired during training and utilized in generalization. Therefore, we employed a representational similarity analysis (RSA) approach (Kriegeskorte et al., 2008; Feng et al., 2021c) to examine the degree of category-related representations applied to each test and whether there are stimulus-related information emerged in aid of generalization.

**Figure 3:**
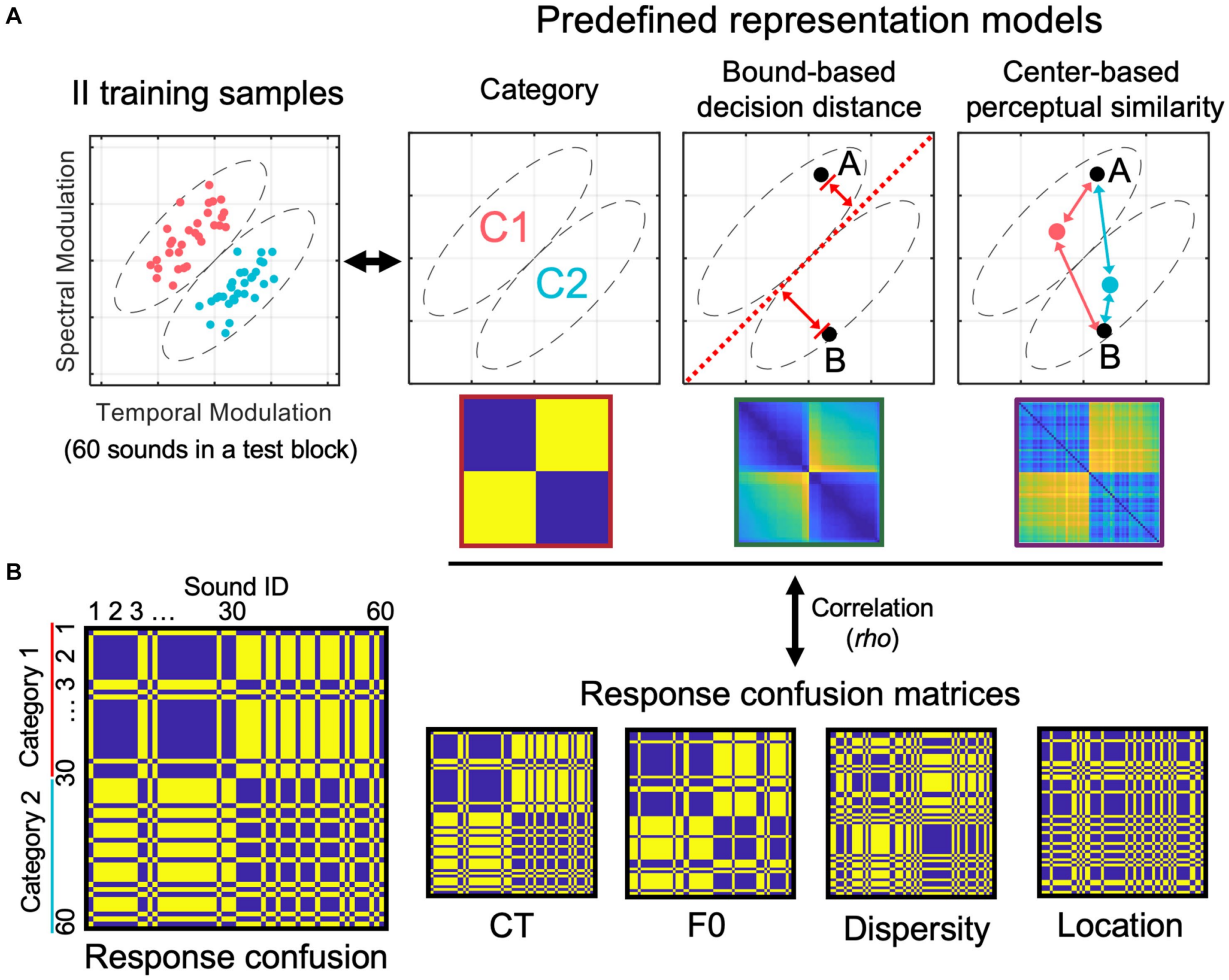
Representational similarity analysis procedure for modeling response confusion patterns to reveal the nature of emergent representations in generalization. **(A)** Three representation models were pre-defined based on category labels, bound-based decision distance, and center-based perceptual similarity. **(B)** Illustration of a response confusion matrix and representative examples of response confusion matrices for the four generalization tests.

We calculated representation similarities between three pre-defined representation models and categorization response confusions produced by individual participants to uncover the nature of emergent representations in generalization. We first constructed three representation models (see Figure 3A) based on binary category labels, bound-based decision distance, and center-based perceptual similarity of the training stimuli, respectively. These models reflect three types of category- or stimulus-related information that the learners could acquire during category learning. These models are operationally defined by representational dissimilarity matrices (RDMs). Each model RDM was fitted with the behavioral response RDMs derived from trial-by-trial categorization responses (Figure 3B).

The category model represents abstract category labels, and the corresponding RDM was designed to have only two dissimilarity values: 0 if two sounds are from the same category or 1 if they are from different categories. For the bound-based decision distance model, to quantify the dissimilarity between each pair of sounds, we used a two-step process. Firstly, we calculated the Euclidean distance between each sound and the optimal bound in the normalized stimulus space, resulting in an *N* × 1 distance array (where *N* is the number of sounds). Secondly, we separated pairs of items into two groups based on their category memberships. For within-category pairs (i.e., items from the same category), we calculated the difference in distance for each pair of sounds to represent dissimilarity. In other words, if two sounds have a similar distance to the bound, they are considered more similar (i.e., have lower dissimilarity) in the RDM. For between-category pairs (i.e., items from different categories), we added up their distances to bound to determine their pairwise dissimilarities. In other words, if two between-category sounds are both far from the bound, this pair has high dissimilarity in the RDM.

To create the center-based perceptual similarity model, we calculated the Euclidean distance between each sound and the center of each category in a 2-dimensional stimulus space. This was done in two steps. Firstly, we calculated two sound-to-category-center distances for each sound item – one for the within-category center and the other for the between-category center (see Figure 3A, the rightmost panel). We then used an equation to convert the Euclidean distance to perceptual similarity (PS):

$$PS_{i}=e^{-D_{i,C}}.$$

C denotes within- or between-category center. $D_{i,C}$ denotes Euclidean distance between sound i and a category center; $e^{-D_{i,C}}$ denotes the PS of sound i to a category center. There are two *PS*s were calculated for each sound item. Secondly, we divided pairs of items into two groups based on their category memberships and then calculated their dissimilarities to construct the model RDM. To represent the dissimilarity of each within-category pair, we computed the average within-category *PS* of two items and subtracted the result from 1. We followed the same method for between-category pairs, averaging two items’ between-category *PS*s and then subtracting the result from 1. The reasoning for this model RDM is that participants could form center-based category representations (i.e., two category centers), and the category representation content of individual items are based on the *PS*s between the items and the two centers (i.e., within- and between-category *PS*s). Based on this model logic, each item’s category representation content can be quantified and compared with other items in a perceptual space, resulting in the center-based perceptual RDM. If this model is supported by the data, we predict that categorization responses are similar (i.e., same response) when two items have similar *PS*s in the space, which results in a high correlation between the model RDM and participants’ response RDMs.

The bound-based decision distance RDM and perceptual similarity RDM were then normalized by scaling between 0 and 1. These model RDMs were constructed based on training samples and applied to fit categorization-response RDMs for each generalization test. Higher model fittings indicate the more robust transfer of the category-and sound-related knowledge acquired in training to the generalization tests.

To calculate the model fit of each model in each generalization test, we first computed a response RDM based on trial-by-trial responses for each block of the tests. Suppose two sounds are responded with the same key, their dissimilarity equal 0, otherwise 1. We sorted items in the response RDM based on their category labels and location in the stimulus space (see Figure 3B for representative response RDMs). To fit the response RDMs, we generated the pre-defined model RDMs based on the sampling distributions of II and RB structures used in training sessions separately. To avoid sampling bias due to a limited number of sounds in each block, we conducted sampling simulations with 10,000 iterations for each model RDM. A model RDM was generated for each simulation and correlated with the response RDMs with Spearman correlation. Average correlation rho was computed for each test and subject. We also examined the unique contribution of each model with a partial correlation approach to control for the variance of the other two models.

#### Simulation and control RSA modeling

To demonstrate the RSA procedure and logic as well as distinctions of the three models, we generated simulated behavioral categorization responses and response confusion matrices based on the model information. We simulated three types of learners based on the knowledge (i.e., binary category, bound-based distance, and center-based PS) they learned and utilized for generalization. To simulate different levels of performances, we also varied the learners’ categorization accuracies across three levels, that is “poor” (mean accuracy = 0.5, range = 0.30–0.75; similar to random responders), “normal” (mean accuracy = 0.80, range = 0.60–0.95; similar to the performances of the participants that we recruited), and “good” (mean accuracy = 0.95, range = 0.80–1). We then generated categorization responses based on binary category, bound-based distance, and center-based PSs information, respectively, to achieve the three levels of performance. We conducted the simulation 10,000 times (i.e., categorized 10,000 items) to generate responses. The responses and errors made by these learners are visualized in Supplementary Figure S2 with a mean accuracy level of 0.8. For the “binary category” learners, errors occurred randomly in the perceptual space for each category. For the “bound-based distance” learners, errors occurred close to the category boundary. For the “center-based perceptual similarity” learners, errors were made based on two item-to-category-center PSs (i.e., within-and between-category PSs). We then converted the simulated responses to behavioral RDMs and conducted RSA to correlate them back to each of the source model RDMs (see Supplementary Figure S2 for detailed simulation results).

To identify participants who may use explicit rules to categorize sounds in the II-structure condition, we conducted control RSA modeling with two additional hypothetical RB models. The two RB models and corresponding RDMs were generated based on the Euclidean distances between each sound and a horizontal or vertical category bound, respectively (refer to non-optimal Uni-X and Uni-Y models; see Supplementary Figure S3 for graphical illustration). We fitted the behavioral response data during training (the last two blocks) and in the control test (CT) for each experiment with the two uni-dimensional bound-based RDMs to identify learners who dominantly used an explicit rule in the II-structure condition. We compared the model fittings of the two non-optimal RDMs with those of the optimal bound-based decision bound RDM (i.e., the RDM created based on the diagonal bound). If a participant’s model fitting either during training or in the CT was higher for the two non-optimal RDMs than the optimal bound-based RDM, we classified those participants as learners who dominantly used RB strategies and removed them from further analyses.

#### Mixed effects modeling

We analyzed participants’ categorization accuracy (ACC), response time (RT), and RSA model fits using linear mixed-effects regression (LMER) analysis. We aim to examine the effects of the generalization test and category structure (II vs. RB learners) on generalization ACC, RT, and model fit for each experiment. The LMER specification of the model was as follows: lmer [Y ~ generalization_test * category_structure + (1|subject)], Y denotes ACC, RT, or RSA model fit. The fixed effects of interest were the category structure and four generalization tests. When collapsed data across the three experiments, training procedure was another fixed factor added into the LMER model to examine the main effect and interaction effects. We also directly compared the effects of generalization tests (i.e., F0 vs. CT; Dispersity vs. CT; Location vs. CT) across the three experiments to examine whether the training procedure modulates generalization performances. The LMER analyses were performed with the software R (version 4.1.0). We analyzed the variances of the LMER models to reveal the statistical significance of the fixed factors and the interaction effects. We used ‘lme4’ package (version 1.1.31) for the mixed-effects modeling. Effect sizes were estimated using the ‘effectsize’ package in R.^1^

## Results

### Learner identification

Across the three experiments, participants in the II condition achieved categorization performances significantly higher than 83% (a benchmark for II learners) at the group level in the immediate CT tests (Exp.1: *M* = 0.863 [*SD* = 0.344]; Exp. 2.1: *M* = 0.875 [*SD* = 0.331]; Exp. 2.2: *M* = 0.894 [*SD* = 0.308]). To identify individual learners who used an explicit rule in the II conditions, we conducted control RSA to model participants’ response data during training (the last two blocks) and the control test (CT) for each experiment with two uni-dimensional bound-based distance models. The two models and their RDMs were generated based on unidimensional rules with horizontal and vertical category bounds, respectively (i.e., non-optimal Uni-X and Uni-Y RDMs, see Supplementary Figure S3 for illustration). We compared the model fittings of the two non-optimal RDMs with those of the optimal bound-based RDM (a diagonal bound) to identify RB learners in the II condition. Those random responders were also identified based on both their categorization accuracy (ACC ≤ 0.53, not significantly better than chance, determined by a permutation test) and model fit (*rho* ≤ 0 for the optimal bound-based distance model). We removed seven participants across all three experiments who dominantly used uni-dimensional bounds (i.e., Uni-X or Uni-Y > optimal bound-based model in model fittings) in the II group (Exp. 1, *N* = 3; Exp. 2.1, *N* = 1; Exp. 2.2, *N* = 3). For the RB group, we conducted similar control RSA modeling to exclude participants who dominantly used non-optimal Uni-X bound to categorize sounds. Eight participants were removed in the RB conditions from further analyses (Exp. 1, *N* = 3; Exp. 2.1, *N* = 4; Exp. 2.2, *N* = 1). After removing those participants, there were 28 participants in Exp. 1 (II group: mean age = 21.07, *SD* = 2.74, *N* = 14; RB group: mean age = 19.40, *SD* = 1.76, *N* = 15), 27 participants in Exp. 2.1 (II group: mean age = 19.60, *SD* = 1.35, *N* = 15; RB group: mean age = 20.17, *SD* = 1.59, *N* = 12), and 29 participants in Exp. 2.2 (II group: mean age = 19.31, *SD* = 1.70, *N* = 13; RB group: mean age = 20.13, *SD* = 2.16, *N* = 16). These six groups of participants were matched in age (chi-square = 9.029, *p* = 0.108) and gender [*F*_(5,79)_ = 1.538, *p* = 0.188] across the three experiments.

### Experiment 1

**Figure 4:**
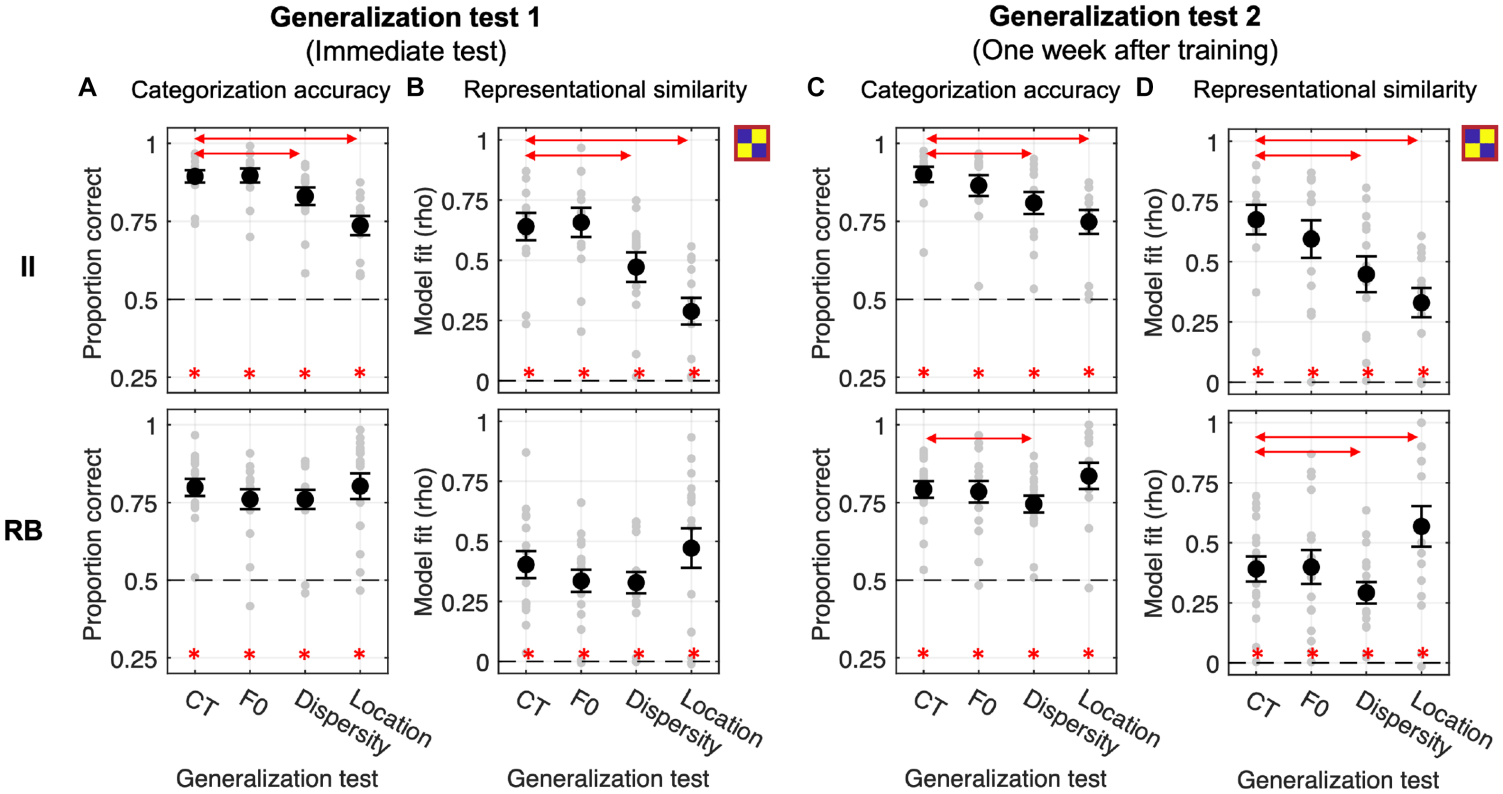
Generalization performances and RSA model fits of the category model across four generalization tests in Exp. 1. **(A,B)** Generalization accuracy and model fit of the immediate test session. **(C,D)** Generalization accuracy and model fit of the test session conducted 1 week after training. Red lines with arrows indicate significant differences between CT and the other three tests. Asterisks represent the above-chance statistical significance (Bonferroni-corrected value of *p*s < 0.05) of each generalization test for each measure.

Both II and RB learners performed significantly above chance (0.5) for the four tests conducted immediately after training at the group level (Bonferroni-corrected *p*s < 0.001, see Figure 4A). For II learners, planned comparisons revealed that the categorization accuracies in Dispersity and Location tests were both significantly lower than CT [Dispersity vs. CT: *t*_(13)_ = −4.405, *p* = 0.002, Cohen *d* = 0.584; Location vs. CT: *t*_(13)_ = −5.186, *p* < 0.001, Cohen *d* = 1.191], while no significant difference was found between F0 and CT [*t*_(13)_ = −0.163, *p* = 1, Cohen *d* = 0.016]. On the contrary, for RB learners, the categorization accuracy was significantly decreased in F0 [*t*_(14)_ = −3.343, *p* = 0.014, Cohen *d* = 0.852], but not in Dispersity [*t*_(14)_ = −2.077, *p* = 0.170, Cohen *d* = 0.449] or Location test [*t*_(14)_ = 0.219, *p* = 1, Cohen *d* = 0.057], compared with that of CT (see detailed pairwise comparisons in Table 1; all value of *p*s from the planned comparisons were Bonferroni-corrected; the same applies hereinafter).

**Table 1:** Planned comparisons between generalization tests in categorization accuracy for each experiment.

Category structure	Generalization test (vs. CT)	*df*	Immediate test			One week after training		
			*t*-value	Corrected *p*-value	Cohen *d*	*t*-value	Corrected *p*-value	Cohen *d*
**Exp. 1 (OT)**								
II	F0	13	−0.163	1	0.016	−1.192	0.764	0.148
	Dispersity	13	−4.405	**0.002**	0.584	−2.523	0.076	0.496
	Location	13	−5.186	**0.001**	1.191	−2.335	0.109	0.597
RB	F0	14	−3.343	**0.014**	0.852	0.256	1	0.035
	Dispersity	14	−2.077	0.17	0.449	−3.389	**0.013**	0.528
	Location	14	0.219	1	0.057	2.352	0.102	0.368
**Exp. 2.1 (CT-NF)**								
II	F0	14	−0.374	1	0.084	0.085	1	0.026
	Dispersity	14	−6.855	**<0.001**	1.004	−2.738	**0.048**	0.682
	Location	14	−5.728	**<0.001**	1.607	−4.839	**0.001**	1.322
RB	F0	11	−0.421	1	0.055	0.542	1	0.11
	Dispersity	11	−3.413	**0.017**	0.564	−1.412	0.557	0.273
	Location	11	1.296	0.664	0.221	0.747	1	0.165
**Exp. 2.2 (CT-WF)**								
II	F0	12	0.107	1	0.034	−2.529	0.079	0.34
	Dispersity	12	−4.729	**0.001**	0.728	−4.595	**0.002**	0.841
	Location	12	−6.407	**< 0.001**	1.681	−5.365	**0.001**	1.323
RB	F0	15	−1.483	0.476	0.319	−0.338	1	0.059
	Dispersity	15	−2.022	0.184	0.329	−3.318	**0.014**	0.437
	Location	15	0.167	1	0.03	1.739	0.307	0.305

The *p*-values were Bonferroni-corrected. The bold values denote significantly above chance (Bonferroni-corrected value of *ps* < 0.05).

The representation modeling of the category model showed similar patterns as the generalization accuracy (see Figure 4B; Table 2). The model fits were all significantly higher than chance (i.e., *rho* > 0) across the four tests and two learner groups (Bonferroni-corrected *p*s < 0.001), indicating category knowledge were transferred to the generalization tests immediately after training. Planned comparisons revealed the same significance patterns as that of the categorization accuracy. For II learners, we found significantly decreased category representations in both Dispersity [*t*_(13)_ = −4.205, *p* = 0.003, Cohen *d* = 0.802] and Location tests [*t*_(13)_ = −4.827, *p* < 0.001, Cohen *d* = 1.356]. For RB learners, we only found significantly decreased category representation in the F0 test [*t*_(14)_ = −3.270, *p* = 0.016, Cohen *d* = 0.699]. No other comparison was found significant (see Table 2 for details).

**Table 2:** Planned comparisons between generalization tests in the model fitting of the category model for each experiment.

Category structure	Generalization test (vs. CT)	*df*	Immediate test			1 week after training		
			*t*-value	Corrected *p*-value	Cohen *d*	*t*-value	Corrected *p*-value	Cohen *d*
**Exp. 1 (OT)**								
II	F0	13	0.323	1	0.044	−1.323	0.626	0.229
	Dispersity	13	−4.205	**0.003**	0.802	−3.728	**0.008**	0.642
	Location	13	−4.827	**0.001**	1.356	−2.649	0.06	0.722
RB	F0	14	−3.27	**0.017**	0.699	1.342	0.603	0.289
	Dispersity	14	−1.485	0.479	0.3	−3.009	**0.028**	0.718
	Location	14	1.154	0.804	0.311	3.235	**0.018**	0.866
**Exp. 2.1 (CT-NF)**								
II	F0	14	0.305	1	0.067	0.383	1	0.111
	Dispersity	14	−6.965	**< 0.001**	1.134	−3.364	**0.014**	0.809
	Location	14	−6.356	**< 0.001**	1.98	−5.769	**< 0.001**	1.332
RB	F0	11	−0.786	1	0.123	0.918	1	0.209
	Dispersity	11	−3.99	**0.006**	0.606	−1.23	0.733	0.303
	Location	11	1.625	0.397	0.272	2.815	0.05	0.71
**Exp. 2.2 (CT-WF)**								
II	F0	12	0.278	1	0.081	−2.17	0.152	0.318
	Dispersity	12	−5.941	**< 0.001**	0.791	−4.956	**0.001**	0.925
	Location	12	−6.617	**< 0.001**	1.742	−7.639	**< 0.001**	1.566
RB	F0	15	−1.545	0.429	0.329	0.197	1	0.031
	Dispersity	15	−2.441	0.083	0.372	−3.839	**0.005**	0.512
	Location	15	1.56	0.419	0.243	4.198	**0.002**	0.632

The *p*-values were Bonferroni-corrected. The bold values denote corrected *p*-values < 0.05.

We conducted the same generalization tests 1 week after training to examine whether the categorization performances and representational properties change over time. For II learners, no planned comparison in accuracy was found significant [F0 vs. CT: *t*_(13)_ = −1.192, *p* = 0.764, Cohen *d* = 0.148; Dispersity vs. CT: *t*_(13)_ = −2.523, *p* = 0.076, Cohen *d* = 0.496; Location vs. CT: *t*_(13)_ = −2.335, *p* = 0.109, Cohen *d* = 0.597] although the overall performance patterns were similar with the immediate tests (Figure 4C). However, the category model fits were significantly decreased in the Dispersity test [Dispersity vs. CT: *t*_(13)_ = −3.728, *p* = 0.008, Cohen *d* = 0.642] but not in the other two tests [Location vs. CT: *t*_(13)_ = −2.649, *p* = 0.060, Cohen *d* = 0.722; F0 vs. CT: *t*_(13)_ = −1.323, *p* = 0.626, Cohen *d* = 0.229]. For the RB learners, the patterns of categorization performances and model fit differed from those found in the immediate tests. Accuracies were significantly decreased only in the Dispersity test [Dispersity vs. CT: *t*_(14)_ = −3.389, *p* = 0.013, Cohen *d* = 0.528] (see Figure 4C; Table 1 for details). The category model fits showed the same decreasing pattern [Dispersity vs. CT: *t*_(14)_ = −3.009, *p* = 0.028, Cohen *d* = 0.718]. However, significantly increased category model fits were found in the Location test [*t*_(14)_ = 3.235, *p* = 0.018, Cohen *d* = 0.866]. These findings suggest that the representational properties of RB categories change over time after training.

### Experiment 2

#### Overall performances during training

During categorization training, participants from both learner groups performed significantly better than chance in training session 2 (see Supplementary Figure S4). Specifically, the categorization accuracy in the last training block (i.e., block 8) was significantly better than chance for both II [Exp. 2.1: *t*_(14)_ = 16.358, *p* < 0.001, Cohen *d* = 5.973; Exp. 2.2: *t*_(12)_ = 28.118, *p* < 0.001, Cohen *d* = 11.028] and RB learners [Exp. 2.1: *t*_(11)_ = 14.568, *p* < 0.001, Cohen *d* = 5.947; Exp. 2.2: *t*_(15)_ = 7.308, *p* < 0.001, Cohen *d* = 2.584]. II and RB learners performed similarly well at the end of training [Exp. 2.1: *t*_(25)_ = −0.245, *p* = 1, Cohen *d* = 0.095; Exp. 2.2: *t*_(27)_ = 1.619, *p* = 0.234, Cohen *d* = 0.117].

#### Generalization performances in Exp. 2.1

**Figure 5:**
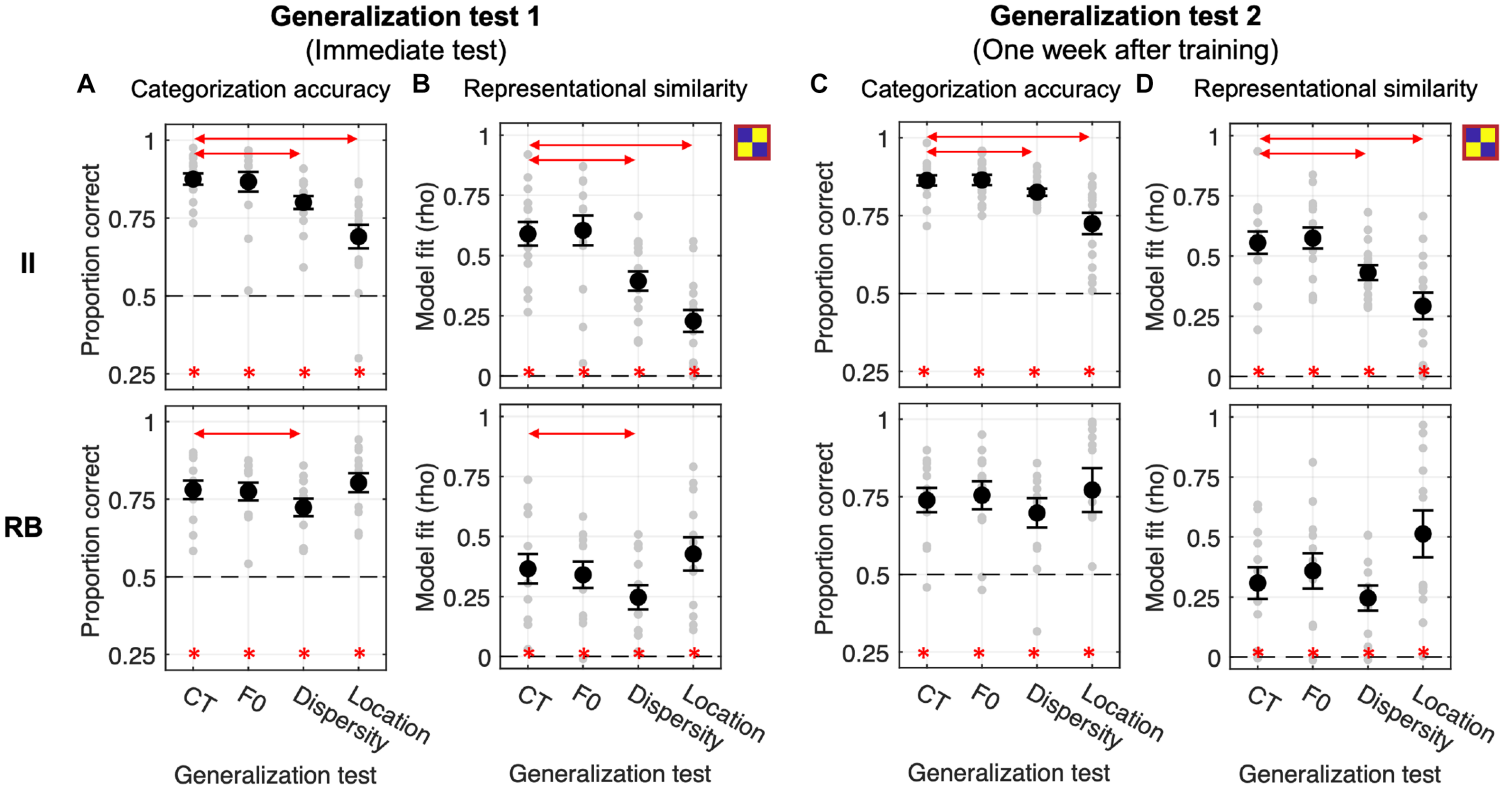
Generalization performances and RSA model fits of the binary category model across four generalization tests in Exp. 2.1 with categorization training without feedback. **(A,B)** Generalization accuracy and model fit of the immediate test session. **(C,D)** Generalization accuracy and model fit of the tests conducted 1 week after training. Red lines with arrows indicate significant differences between CT and the other three tests. Asterisks represent the above-chance statistical significance (Bonferroni-corrected value of *p*s < 0.05) of each generalization test for each measure.

This experiment examines whether categorization training without feedback could modulate the effect of generalization (i.e., F0/Dispersity/Location vs. CT). As shown in Figure 5A, both learner groups performed significantly above chance in the four tests conducted immediately after training (all Bonferroni-corrected *p*s < 0.01). For the II learners, planned comparisons showed significantly decreased categorization accuracy in Dispersity [*t*_(14)_ = −6.855, *p* < 0.001, Cohen *d* = 1.004] and Location tests [*t*_(14)_ = −5.728, *p* < 0.001, Cohen *d* = 1.607] compared to CT. Changes in F0 did not affect generalization [F0 vs. CT: *t*_(14)_ = −0.374, *p* = 1, Cohen *d* = 0.084]. Changes in sampling dispersity also affected generalization performances for RB learners [Dispersity vs. CT: *t*_(11)_ = −3.413, *p* = 0.017, Cohen *d* = 0.564], whereas the other two factors did not affect generalization [F0 vs. CT: *t*_(11)_ = −0.421, *p* = 1, Cohen *d* = 0.055; Location vs. CT: *t*_(11)_ = 1.296, *p* = 0.664, Cohen *d* = 0.221]. These generalization patterns were similar to those found in Exp. 1.

The category model fits were significantly better than chance across tests and learner groups (Figure 5B). For II learners, similar to the accuracy data, we found significantly decreased category model fits in Dispersity and Location tests compared to CT [Dispersity vs. CT: *t*_(14)_ = −6.965, *p* < 0.001, Cohen *d* = 1.134; Location vs. CT: *t*_(14)_ = −6.356, *p* < 0.001, Cohen *d* = 1.980]. Increased sampling dispersity also significantly affected category representations for RB learners [Dispersity vs. CT: *t*_(11)_ = −3.990, *p* = 0.006, Cohen *d* = 0.606]. No other contrasts between tests reached significance (see Table 2 for details).

Generalization tests performed 1 week after training were all significantly above chance across learner groups (Figure 5C). Similar to the immediate tests, for II learners, changes in sampling dispersity and locations in the perceptual space significantly affected generalization performances [Dispersity vs. CT: *t*_(14)_ = −2.738, *p* = 0.048, Cohen *d* = 0.682; Location vs. CT: *t*_(14)_ = −4.839, *p* < 0.001, Cohen *d* = 1.322], but not for the F0 changes [F0 vs. CT: *t*_(14)_ = 0.085, *p* = 1, Cohen *d* = 0.026]. In contrast, RB learners’ generalization performances were not affected by these factors [F0 vs. CT: *t*_(11)_ = 0.542, *p* = 1, Cohen *d* = 0.110; Dispersity vs. CT: *t*_(11)_ = −1.412, *p* = 0.557, Cohen *d* = 0.273; Location vs. CT: *t*_(11)_ = 0.747, *p* = 1, Cohen *d* = 0.165], which was different from the patterns of immediate tests.

The representation modeling results are consistent with the generalization accuracy findings (see Figure 5D; Table 2): II learners, Dispersity vs. CT [*t*_(14)_ = −3.364, *p* = 0.014, Cohen *d* = 0.809); Location vs. CT (*t*_(14)_ = −5.769, *p* < 0.001, Cohen *d* = 1.332]. No other comparison was found significant.

#### Generalization performances in Exp 2.2

In this experiment, we aim to examine to which extent categorization training with feedback facilitates category learning and modulates the effects of generalization. However, we did not find a significant difference in categorization performances between Exp. 2.1 and Exp. 2.2 at the end of training [II learners: *t*_(26)_ = 1.653, *p* = 0.110, Cohen *d* = 0.626; RB learners: *t*_(26)_ = −0.817, *p* = 0.421, Cohen *d* = 0.312] and in the immediate CT [II learners: *t*_(26)_ = 0.720, *p* = 0.478, Cohen *d* = 0.273; RB learners: *t*_(26)_ = 0.456, *p* = 0.652, Cohen *d* = 0.174].

**Figure 6:**
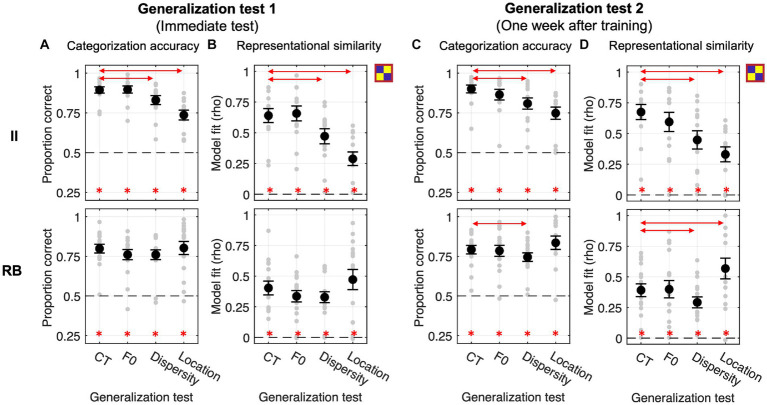
Generalization performances and RSA model fits of the category model across four generalization tests in Exp. 2.2 with feedback-based categorization training. **(A,B)** Generalization accuracy and model fit of the immediate test session. **(C,D)** Generalization accuracy and model fit of the tests conducted 1 week after training. Red lines with arrows indicate significant differences between CT and the other three tests. Asterisks denote above-chance significance (Bonferroni-corrected value of *p*s < 0.05).

In the immediate tests, II and RB learners performed significantly above chance across tests (Figure 6A, all Bonferroni-corrected *p*s < 0.001). For II learners, similar to the Exp. 2.1, categorization accuracy and category model fits were both significantly decreased in Dispersity [accuracy: *t*_(12)_ = −4.729, *p* = 0.001, Cohen *d* = 0.728; model fit: *t*_(12)_ = −5.941, *p* < 0.001, Cohen *d* = 0.791] and Location tests [accuracy: *t*_(12)_ = −6.407, *p* < 0.001, Cohen *d* = 1.681; model fit: *t*_(12)_ = −6.617, *p* < 0.001, Cohen *d* = 1.742] compared to CT. For RB learners, no planned comparison in accuracy or model fit reached significance (see Tables 1, 2 for details).

For the generalization tests performed 1 week after training, categorization accuracies and model fits of the category model were all significantly better than chance across learner groups (Figures 6C,D). Similar to the immediate test, II learner’s categorization accuracies and model fits were significantly decreased in Dispersity [accuracy: *t*_(12)_ = −4.595, *p* = 0.002, Cohen *d* = 0.841; model fit: *t*_(12)_ = −4.956, *p* < 0.001, Cohen *d* = 0.925] and Location tests [accuracy: *t*_(12)_ = −5.365, *p* < 0.001, Cohen *d* = 1.323; model fit: *t*_(15)_ = −7.639, *p* < 0.001, Cohen *d* = 1.566] compared to CT. For RB learners, categorization accuracies and model fits were both affected by the dispersity changes [accuracy: Dispersity vs. CT: *t*_(15)_ = −3.318, *p* = 0.014, Cohen *d* = 0.437; model fit: *t*_(15)_ = −3.839, *p* = 0.005, Cohen *d* = 0.512], which is different from the immediate generalization. In contrast, the model fits were significantly increased in Location compared to CT [*t*_(15)_ = 4.198, *p* = 0.002, Cohen *d* = 0.632]. These findings indicated better category knowledge transferred in Location, but worse in Dispersity conditions. No other planned comparison reached significance (see Tables 1, 2 for details).

### Statistical summaries across the three experiments

The above results reported for each experiment highlighted the effects of generalization (i.e., F0/Dispersity/Location vs. CT) and distinct patterns of these effects for II and RB learners. To directly compare generalization effects across learner groups, experiments (i.e., training procedures), and types of generalization, we used CT as a baseline to compute the generalization effects induced by changes in F0, sampling dispersity, and location in the perceptual space. The three types of generalization effects (i.e., F0 – CT; Dispersity – CT; Location – CT) were calculated for each learner, experiment, and test, which enables the direct comparisons in generalization effects between learner groups (II vs. RB), generalization types (F0 vs. Dispersity vs. Location), and training procedures (OT vs. CT-NF vs. CT-WF) even though the baseline categorization performances differed across conditions. A three-factor LMER was conducted to reveal the main effects of learner group, generalization type, training procedure, and their interaction effects on the generalization effects.

**Figure 7:**
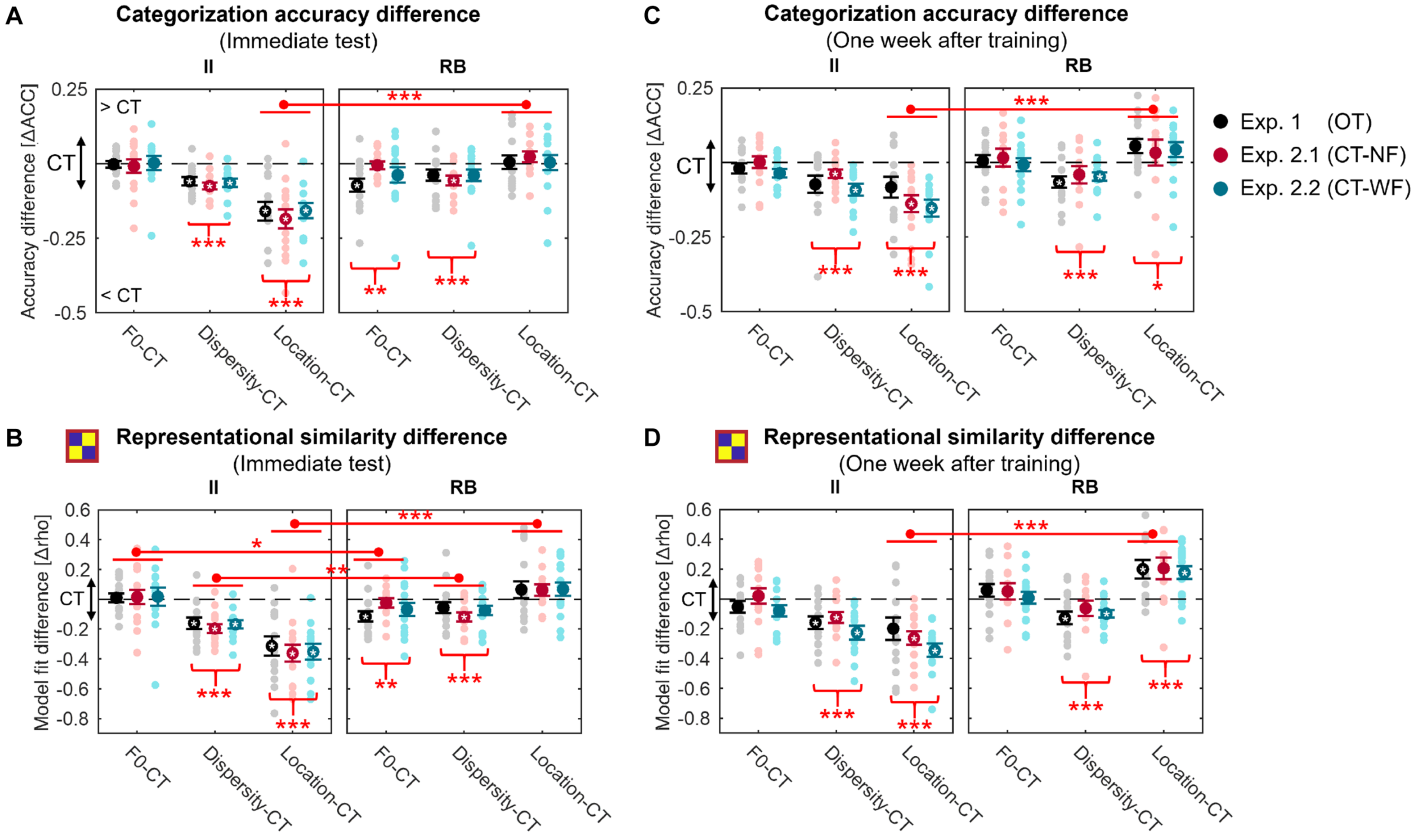
Changes in generalization accuracy **(A,C)** and RSA model fits of the binary category model **(B,D)** for the generalization tests performed immediately **(A,B)** and 1 week after training **(C,D)** for the three experiments. Red asterisks under the curly brackets denote significant differences between CT and the other three tests, with data collapsed across the three experiments. Red lines denote significant differences between II and RB learners, with data collapsed across all experiments. White asterisks denote significant differences between CT and each of the other three tests for each experiment. *p < 0.05; **p < 0.01; **p < 0.001; corrected *p* values.

For the immediate generalization performances (Figure 7A), we found significant main effects of learner group [*F*_(1, 85)_ = 16.431, *p* < 0.001, partial *η*^2^ = 0.16] and generalization type [*F*_(2, 170)_ = 20.584, *p* < 0.001, partial *η*^2^ = 0.19], and a significant learner-group-by-generalization-type interaction effect [*F*_(2, 170)_ = 74.694, *p* < 0.001, partial *η*^2^ = 0.47] indicating distinct patterns of the generalization effects between II and RB learners. No other effects were found significant. Similarly, for category model fits (Figure 7B), we found significant main effects of learner group [*F*_(1, 85)_ = 27.434, *p* < 0.001, partial *η*^2^ = 0.24] and generalization type [*F*_(2, 170)_ = 19.829, *p* < 0.001, partial *η*^2^ = 0.19], and a significant learner-group-by-generalization-test interaction effect [*F*_(2, 170)_ = 81.029, *p* < 0.001, partial *η*^2^ = 0.49]. Since neither the main effect of training procedure nor any interaction effect related to training procedure was found significant, we conducted *ad hoc* comparisons with data collapsed across the three experiments.

Consistent with the results reported in each experiment, changes in sampling dispersity and location in the perceptual space significantly affected generalization performances for II learners [accuracy: Dispersity vs. CT, *t*_(41)_ = −9.253, *p* < 0.001, Cohen *d* = 2.019; Location vs. CT, *t*_(41)_ = −9.937, *p* < 0.001, Cohen *d* = 2.169; category model fit: Dispersity vs. CT, *t*_(41)_ = −9.676, *p* < 0.001, Cohen *d* = 2.112; Location vs. CT, *t*_(41)_ = −10.294, *p* < 0.001, Cohen *d* = 2.246]. However, changes in F0 did not affect generalization performances [accuracy: *t*_(41)_ = −0.248, *p* = 1, Cohen *d* = 0.054; category model fit: *t*_(41)_ = 0.514, *p* = 1, Cohen *d* = 0.112]. For RB learners, categorization accuracy and the robustness of category representations in generalization were significantly affected by the changes in F0 [accuracy: *t*_(42)_ = −3.128, *p* = 0.010, Cohen *d* = 0.675; category model fit: *t*_(42)_ = −3.222, *p* = 0.007, Cohen *d* = 0.695] and sampling dispersity [accuracy: *t*_(42)_ = −4.153, *p* < 0.001, Cohen *d* = 0.896; category model fit: *t*_(42)_ = −4.180, *p* < 0.001, Cohen *d* = 0.901].

Furthermore, the generalization effect of Location (i.e., Location vs. CT) in categorization accuracy (Figure 7A) was more salient for II than RB learners [*t*_(83)_ = −8.372, *p* < 0.001, Cohen *d* = 1.816], suggesting that changes in location of the perceptual space affected generalization of II category knowledge more than that of RB. For category model fits (Figure 7B), generalization effects of sampling dispersity [*t*_(83)_ = −3.589, *p* = 0.002, Cohen *d* = 0.779] and location of perceptual space [*t*_(83)_ = −9.529, *p* < 0.001, Cohen *d* = 2.067] were both more salient for II learners than that of RB. In contrast, the generalization effect of F0 was more robust for RB than II learners [*t*_(83)_ = 2.469, *p* = 0.047, Cohen *d* = 0.536].

Generalization effects were similar between the immediate tests and tests conducted 1 week after training (Figures 7C,D). Two intriguing differences were found between the tests conducted a week separately. Firstly, the generalization effect of F0 was not significant for RB learners 1 week after training, neither for accuracy [*t*_(42)_ = 0.266, *p* = 1, Cohen *d* = 0.058] nor category model fits [*t*_(42)_ = 1.450, *p* = 0.463, Cohen *d* = 0.313], which may indicate of a category representation transformation or abstraction after training. The second difference is that both accuracy and category model fits in Location for the RB learners were significantly higher than that in CT 1 week after training [accuracy: *t*_(42)_ = 2.624, *p* = 0.036, Cohen *d* = 0.569; category model fit: *t*_(42)_ = 5.892, *p* < 0.001, Cohen *d* = 1.271], which may indicate the increasing transfer of category knowledge to sounds from the untrained location of perceptual space during a week after training.

We further examined what stimulus- or category-related knowledge was represented and utilized in generalization and to which extent these representations changed due to the changes in F0, sampling dispersity, and locations of perceptual space. Thus, we used three predefined representation models (i.e., category, bound-based decision distance, and center-based perceptual similarity models) to fit learners’ response RDMs and compared the model fits between CT and the other three tests to reveal the representation changes.

**Figure 8:**
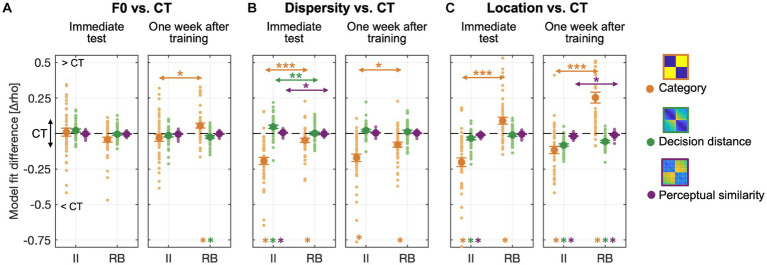
Representation modeling of categorization confusion matrices with three predefined representation models using data collapsed across the three experiments. The unique contribution of each model was calculated and subtracted from CT to reveal the generalization effects. **(A)** RSA Model fit differences between F0 and CT tests for both groups; **(B)** Model fit differences between Dispersity and CT tests; **(C)** Model fit differences between Location and CT tests. Colored asterisks at the bottom of each graph denote significant differences between CT and each of the other three tests. Arrow lines denote significant differences between II and RB learners in model fit (*p < 0.05; **p < 0.01; **p < 0.001; corrected *p* values).

For all the generalization tests, each of the three representation models was significantly correlated with learners’ response RDMs (Supplementary Figure S5), indicating that these models all contribute to explaining learners’ categorization response patterns. The category model explained the most variances among the three models across all tests except for the Dispersity tests. We further calculated the unique contribution of each model in explaining the response confusion patterns by controlling for the variances of the other two models using the partial correlation approach and compared these model fits between tests and learners (Figure 8).

For generalization tests performed immediately after training, the F0 change did not affect the transfer of the three types of category-related representations for both learner groups (Figure 8A, left panel). However, RB learners’ category representations increased 1 week after training in the F0 test (Figure 8A, right panel) and were significantly higher than CT [*t*_(42)_ = 3.264, *p* = 0.007, Cohen *d* = 0.704]. This increase in category representations was accompanied by a decrease in the representation of decision-bound distance [*t*_(42)_ = −2.869, *p* = 0.019, Cohen *d* = 0.619].

Compared to CT, increased sampling dispersity in the Dispersity test affected category representations for both II and RB learners in tests conducted both immediately and 1 week after training [Figure 8B; II learners: immediate test, *t*_(41)_ = −7.685, *p* < 0.001, Cohen *d* = 1.677; 1 week after training, *t*_(41)_ = −6.532, *p* < 0.001, Cohen *d* = 1.425; RB learners: immediate test, *t*_(42)_ = −3.128, *p* = 0.010, Cohen *d* = 0.675; 1 week after training, *t*_(42)_ = −4.467, *p* < 0.001, Cohen *d* = 0.963]. More decrease in category representations was found for II than RB learners [immediate test, *t*_(83)_ = −4.956, *p* < 0.001, Cohen *d* = 1.075; one week after training: *t*_(83)_ = −2.956, *p* < 0.012, Cohen *d* = 0.641]. In the immediate test, the decreased category representations was accompanied by increased representations of decision distance [*t*_(41)_ = 4.011, *p* < 0.001, Cohen *d* = 0.875] and perceptual similarity [*t*_(41)_ = 2.539, *p* < 0.045, Cohen *d* = 0.554] only for II learners, but not for RB learners [decision distance model: *t*_(42)_ = 0.032, *p* = 1, Cohen *d* = 0.007; perceptual similarity model: *t*_(42)_ = −1.321, *p* = 0.581, Cohen *d* = 0.285]. More increased representations of decision distance [*t*_(83)_ = 3.101, *p* = 0.008, Cohen *d* = 0.673] and perceptual similarity [*t*_(83)_ = 2.764, *p* = 0.021, Cohen *d* = 0.600] were found for II than RB learners.

Changes in locations of perceptual space in Location test modulated the degrees of category representations for both II and RB learners, but in opposite directions [Figure 8C; II, immediate test: *t*_(41)_ = −6.701, *p* < 0.001, Cohen *d* = 1.462; 1 week after training: *t*_(41)_ = −4.469, *p* < 0.001, Cohen *d* = 0.975; RB, immediate test: *t*_(42)_ = 3.670, *p* = 0.002, Cohen *d* = 0.791; 1 week after training: *t*_(42)_ = 6.552, *p* < 0.001, Cohen *d* = 1.413]. For II learners, decreased category representations were accompanied by decreased representations of decision distance [immediate test: *t*_(41)_ = −3.518, *p* = 0.003, Cohen *d* = 0.768; 1 week after training: *t*_(41)_ = −9.753, *p* < 0.001, Cohen *d* = 2.128] and perceptual similarity [immediate test: *t*_(41)_ = −5.539, *p* < 0.001, Cohen *d* = 1.209; 1 week after training: *t*_(41)_ = −7.162, *p* < 0.001, Cohen *d* = 1.563]. For RB learners, increased category representations were accompanied by decreased representations of decision distance [*t*_(40)_ = −6.969, *p* < 0.001, Cohen *d* = 1.539] and perceptual similarity (*t*_(40)_ = −3.337, *p* = 0.006, Cohen *d* = 0.737) only for the tests performed 1 week after training. More decreased representations of perceptual similarity were found for II than RB learners 1 week after training [*t*_(81)_ = −2.470, *p* = 0.047, Cohen *d* = 0.542].

Regarding the categorization response time (RT, see Supplementary Figure S6), we found a significant main effect of training procedure [*F*_(2,85)_ = 7.242, *p* = 0.001, partial *η*^2^ = 0.15]. Faster categorization responses were shown in Exp. 2.2 [CT-WF vs. OT: *F*_(1, 58)_ = 8.734, *p* = 0.005, partial *η*2 = 0.13; CT-WF vs. CT-NF: *F*_(1, 56)_ = 12.241, *p* < 0.001, partial *η*2 = 0.18]. This training procedure effect is independent of category structures, suggesting that categorization training with feedback facilitates categorization decisions in generalization contexts in general. We also found a significant main effect of generalization test [*F*_(3, 595)_ = 3.706, *p* = 0.012, partial *η*^2^ = 0.02] where learners required a longer time to categorize in the Dispersity test [Dispersity vs. CT: *F*_(1, 255)_ = 8.041, *p* = 0.005, partial *η*^2^ = 0.03; F0 vs. CT: *F*_(1, 255)_ = 0.008, *p* = 0.927, partial *η*^2^ < 0.001; Location vs. CT: *F*_(1, 255)_ = 1.424, *p* = 0.234, partial *η*^2^ < 0.001]. We did not find a significant main effect of leaner group [*F*_(1,85)_ = 2.330, *p* = 0.131, partial *η*^2^ = 0.03] but found a significant learner-by-test interaction effect [*F*_(3,595)_ = 7.392, *p* < 0.001, partial *η*^2^ = 0.04]. For II learners, the main effect of generalization test was significant [*F*_(3, 294)_ = 7.882, *p* < 0.001, partial *η*^2^ = 0.07], where Dispersity and Location tests responded significantly longer than other tests [F0 vs. CT: *F*_(1, 126)_ = 0.837, *p* = 0.362, partial *η*^2^ < 0.001; Dispersity vs. CT: *F*_(1, 126)_ = 9.086, *p* = 0.003, partial *η*^2^ = 0.07; Location vs. CT: *F*_(1, 126)_ = 8.385, *p* = 0.004, partial *η*^2^ = 0.06]. In contrast, for RB learners, the main effect of generalization test failed to reach significance [*F*_(3, 301)_ = 2.309, *p* = 0.077, partial *η*2 = 0.02].

## Discussion

Learners’ ability to generalize to novel testing situations directly speaks to the nature of representations acquired in training and the mechanisms supporting the cross-situation transfer of category knowledge. Here, with this experimental logic, we designed four generalization tests with different types of novel items and conducted three category learning experiments with different training procedures to probe the differences in representation nature between II and RB categories. Across three experiments, we found distinct characteristics of the newly-acquired II and RB category representations indicated by generalization patterns and representation modeling. II and RB learners transferred category knowledge to the four tests to different extents. The II learners significantly decreased generalization performances and category representations in the Dispersity and Location tests but not in the F0 test compared to the baseline test, which suggests that the generalization of II category knowledge is not affected by surface acoustic changes in an irrelevant F0 dimension but less successful in categorizing new items sampled from an untrained perceptual area (i.e., the Location test) and in a context with more disperse samples (i.e., the Dispersity test). In contrast, RB learners’ generalizations are resistant to changes in perceptual regions (i.e., the Location test) and interference of the F0 change (especially for the representations after stabilization, i.e., 1 week after training), but they are sensitive to changes in increasing dispersity of exemplars (i.e., the Dispersity test). Representational similarity modeling further revealed that II and RB learners used different generalization mechanisms in the Dispersity test. II learners enhanced representations of perceptual similarity and decision distance to compensate for the decreased transfer of category-label representations, whereas RB learners used a more computational cost mechanism by default, computing the decision-bound distance to guide categorization decisions. These findings provide new insights into understanding the relationships between category learning, the nature of category representations, and how different learning and representation systems operate differently to achieve successful generalization.

We aim to examine the representation nature of II and RB categories, especially the abstractness of the category representations utilized in generalization. We manipulated three factors in generalization, ranging from low-level surface acoustics to higher-level exemplar dispersity and untrained perceptual location. Based on DLS models, especially the COVIS model in the visual domains, II representations are tightly linked to the exemplar-dependent or feature-specific information (e.g., visual field), and any changes in the low-level information affect generalization performances (Ashby et al., 2003; Maddox et al., 2004b, 2005, 2007; Rosedahl et al., 2018). However, across the three experiments, we demonstrate that II learners can resist changes in F0 for generalization tests performed immediately and 1 week after training, suggesting that auditory II category representations are not tightly linked to low-level dimensions irrelevant to discriminating category members. Changes in F0 analog changes in talker (e.g., Bradlow and Bent, 2008; Xie et al., 2021). This finding is consistent with previous auditory and speech category learning studies that showed talker-independent category representations emerged at both behavioral and neural levels during training (Bradlow and Bent, 2008; Feng et al., 2019, 2021c; Johnson and Sjerps, 2021; Xie et al., 2021). In speech perception, native listeners can maintain perceptual constancy while facing various types of acoustic and perceptual variabilities (Bradlow and Bent, 2008; Feng et al., 2018, 2021a), suggesting exceptional generalization ability to overcome the “lack-of-invariance” challenge.

In comparison, RB learners’ generalization performances were not consistently affected by F0 changes. The generalization performances in the immediate F0 test were significantly less accurate than the control test, especially for Exp. 1 with observational training. However, the generalization was insensitive to F0 changes 1 week after training across the three experiments. These findings suggest that RB learners may undergo a transformation or abstraction process within a week after training to stabilize the category representations to overcome the variability induced by an irrelevant acoustic dimension. One speculation is that this post-training abstraction or stabilization process is specific for RB categories, where the F0 information could be efficiently integrated into the previously-acquired categorization rules so that the irrelevant acoustic information is eliminated before the decision (e.g., application of new filtering rules). Adopting a new rule to filter irrelevant information while maintaining attention to the relevant dimensions or features may require time and the support of executive systems (Diekelmann and Born, 2010; Genzel et al., 2014). The distinct patterns between II and RB learners in the F0 test suggest that the category structure we learn plays an important role in how the auditory perception system copes with novel acoustic variabilities.

One of the critical tests for the abstractness of emerged representations is to test the generalization to new sounds sampled from an untrained area of the stimulus space. We found that significantly reduced generalization performances and decreased category model fittings to new sounds outside the range of training samples (i.e., in the new location test) for II learners across the three experiments. In contrast, increased generalization performances and enhanced category representations for the RB learners were found in the same test. This dissociated pattern suggests that II representations are tightly restricted to the perceptual areas where the training samples are covered. The trained locations in the stimulus space may be weighted differently than untrained areas, so the categorization performances are compromised when the category knowledge is transferred from trained locations to untrained locations.

These results are consistent with previous findings from studies of visual category learning. For example, Smith et al. (2015) asked two groups of participants to learn RB and II categories, respectively, and required them to categorize new stimuli sampled from an untrained region of the stimulus space. They found that RB learners’ generalization was nearly seamless, whereas II learners failed to categorize the new items. Moreover, II representations are often linked with exemplar-associated sensorimotor features. Changes in the visual field have significant interferences of II categorization but not RB (Rosedahl et al., 2018), suggesting visual-field-dependent representations of II categories. Similarly, changing the response key to categorize items only affects the II categorization but not RB (Ashby et al., 2003), suggesting that visual II categories are tightly associated with their motor correspondences. Comparing these findings from visual domains, our findings in auditory category learning indicate that II auditory category representations are only tightly linked to the perceptual properties of the dimensions relevant to categorization instead of any other irrelevant dimensions (e.g., F0) inherent from the training samples. This representation mechanism of II auditory categories may help listeners precisely generalize category knowledge to novel items and scenarios with redundant and irrelevant acoustic information.

Another critical test of the representation nature is to examine the generalization performances to new sounds with distinct distributional patterns from the training samples. We found that reduced categorization accuracy and decreased category representations when learners generalized to a set of new sounds with a more dispersed distribution (i.e., the Dispersity test) compared to the training samples for both II and RB learners. These findings indicate that both II and RB category representations are sensitive to the within-category sound distributional pattern. DLS models predict that learning II and RB categories recruit distinct learning strategies and neurocognitive systems (Ashby and Maddox, 2005, 2011; Chandrasekaran et al., 2014a,b). However, DLS models do not have detailed descriptions and predictions about whether the category representations are associated with the within-category distributional statists of the training samples. DLS model may predict that the generalization of II category representations is affected by changes in sound dispersity because II category representations are hypothesized to link with sample-specific features. This reasoning is supported by recent findings that II but not RB categories with a probabilistic distribution are learned worse than categories with a deterministic distribution (Roark and Holt, 2018), suggesting II category representations are formed based on or along with distributional information. Any changes in the stimulus distributional pattern would affect II generalization performances. Even though the overall coverage in the perceptual space is the same for the training and generalization samples, the distributional patterns within each category are different. The training samples are normally distributed in the space (i.e., center-based distribution pattern), whereas the generalization samples in the Dispersity test are more distributed. Thus, our results suggest that forming II category representations is a learning process that considers all types of variabilities in the perceptual space. Any changes in sample distribution or the locations in the perceptual space, whether within or outside the coverage of training samples, would affect the transfer of the II category knowledge.

A perceptual weighting mechanism may explain the II generalization decreases in the Dispersity and Location tests (Niv et al., 2015). Successful learning of II auditory categories requires integrating information from the spectral- and temporal-modulation dimensions. The integration ratio of the two dimensions is subject to the distributions of training samples. The distribution of the training samples provides statistical information about the category probabilities for the learners to weigh the two dimensions. Learners may gradually adjust the integration ratio or weights of the two dimensions as they expose to more samples. Higher perceptual weights would be assigned to the perceptual areas more frequently encountering samples. Transferring this weighting knowledge from trained areas to untrained areas (e.g., in the Location test) or from more trained areas to less trained areas (e.g., in the Dispersity test) in the stimulus space would be less successful because these weighting representations are acquired with the procedure-based learning system and tapped into the auditory cortex. In addition, sounds from untrained perceptual areas may not be weighted the same way in the stimulus space as the trained sounds, which may lead to incorrect categorization. This speculation is consistent with our recent neuroimaging findings. Feng et al. (2021a,b,c) examined the neural encodings of II and RB auditory categories during categorization training. They found that auditory and speech perception-related regions in the superior temporal cortex gradually increased the weightings of perceptual information for II sounds. In contrast, the same regions gradually decreased the perceptual representations for RB sounds. This putative perceptual weighting mechanism of II representations requires further examination.

We found that changes in sampling dispersity, rather than perceptual locations, affect the ability to categorize novel RB sounds. These findings may contradict predictions made by DLS models and recent studies (Maddox et al., 2005; Roark and Holt, 2018). RB category representations are considered to be abstract rules associated with executive functions and working memory capacity (Waldron and Ashby, 2001; Zeithamova and Maddox, 2006; DeCaro et al., 2008; Reetzke et al., 2016). The factor of sampling dispersity is not explicitly linked to these functions. One possibility is that categorization rules are derived from the probability distribution of training samples. Learners may use the knowledge of a probability distribution around the decision boundary learned during training to apply rules to categorize new RB sounds. Our representational similarity modeling results show that the bound-based distance model significantly correlates with categorization confusions (see Supplementary Figure S5), suggesting that the bound-based sample probability information is a necessary representation for generalization to more dispersed contexts. If the new RB sounds have different sampling dispersity, this can lead to the transfer of these probabilities and rules being less accurate, thus leading to a decrease in categorization performance.

It is also possible that RB-specific generalization mechanisms play a role in learners’ ability to categorize novel RB sounds. When encountering new RB sounds, learners may estimate the bound-based distance to assist with categorization. This may be especially true when there is no previous bound-based distance information can be retrived to aid in generalization. Computing bound-based distance in real-time for generalizations can be a computational cost strategy as it requires effort and processing resources. The accuracy of computing the sound-to-boundary distance may be related to the sampling dispersity. When there are more distributed sounds within each category, there may be a less accurate estimation of the bound distance between each sound and a putative category bound. This can affect the categorization decision in the Dispersity test compared to the control test. Further studies are needed to investigate these possibilities systematically.

Regarding the training procedure, we did not find any significant differences in generalization accuracy or representational model fit across the three experiments. In addition, the training procedure did not affect the differences between II and RB in generalization performances. These findings suggest that categorization tasks and providing immediate feedback compared to the observation training may not additionally facilitate learning and generalization accuracies for both category structures. In contrast to the generalization accuracy and representation modeling, we found that categorization training with feedback (i.e., Exp. 2.2) facilitated learners’ categorization response time across generalization tests (Supplementary Figure S6), while this facilitation is not a tradeoff of categorization accuracies. These findings indicate that categorization training with feedback may facilitate learners’ categorization decision processes, such as more efficiency in accumulating information for category decisions compared to the observation training. Providing feedback and presenting category labels may play a similar role for RB’s hypothesis-testing and II’s associative learning processes, except that feedback-based training recruits reinforcement learning mechanisms to learn sound-to-category mappings (Ashby and Maddox, 2005). The feedback-dependent reinforcement learning may facilitate the formation of many-to-one corticostriatal projections from the auditory and prefrontal cortex to the striatum regions (Chandrasekaran et al., 2014a,b; Maddox and Chandrasekaran, 2014; Feng et al., 2021b). The reinforcement-based mechanism may facilitate the retrieval of the category knowledge acquired during feedback-based categorization training, thus enhancing the decision processing in categorization. More investigations are needed to test this possibility.

## Summary and conclusion

We examined the nature of II and RB auditory category representations by accessing the ability of learners to generalize new category knowledge to novel testing situations. Four generalization tests with various novel items were designed, and three category learning experiments with different training procedures were conducted to compare the characteristics of newly acquired II and RB category knowledge. II learners demonstrated decreased generalization performance and category representation when generalizing to untrained perceptual areas and in contexts with more dispersed samples; on the other hand, RB learners showed resistance to changes in perceptual regions but sensitivity to increasing dispersity of sounds. II and RB learners used different strategies for generalization when facing the more variable context, with II learners enhancing representations of perceptual similarity and decision distance while RB learners employed a more computational cost strategy to guide categorization decisions. These findings shed light on the relationship between category learning and representation nature and the varying strategies used by different learning and representation systems to achieve successful generalization.

## Data availability statement

The raw data supporting the conclusions of this article will be made available by the authors, without undue reservation.

## Ethics statement

The studies involving human participants were reviewed and approved by the ethics review board of the School of Psychology at South China Normal University and the Joint Chinese University of Hong Kong – New Territories East Cluster Clinical Research Ethics Committee. The participants provided their written informed consent to participate in this study.

## Author contributions

ZG: data curation, project administration, and writing - original draft preparation. LZ: investigation. SW: project administration and funding acquisition. GF: Conceptualization, data curation, methodology, writing - original draft preparation, writing – reviewing and editing, supervision, and funding acquisition.

## Funding

The work described in this paper was supported by grants from the Research Grants Council of the Hong Kong Special Administrative Region, China (Project No.: 14614221, 14612923, and 14619518 to Gangyi Feng) and the National Natural Science Foundation of China (Project No. 32171051 to SW).

## Conflict of interest

The authors declare that the research was conducted in the absence of any commercial or financial relationships that could be construed as a potential conflict of interest.

## Publisher’s note

All claims expressed in this article are solely those of the authors and do not necessarily represent those of their affiliated organizations, or those of the publisher, the editors and the reviewers. Any product that may be evaluated in this article, or claim that may be made by its manufacturer, is not guaranteed or endorsed by the publisher.

## Footnotes

^1^ https://easystats.github.io/effectsize/
