# Supplementary material for: Distribution-dependent representations in auditory category learning and generalization
Source: Front Psychol. 2023 Sep 27;14:1132570. doi: 10.3389/fpsyg.2023.1132570 (PMC10566369; doi:10.3389/fpsyg.2023.1132570)
Supplement: Supplementary file 1 [file Data_Sheet_1.docx]

**Supplementary Results**

**Categorization simulations based on the three pre-defined models**

To demonstrate the RSA procedure and analysis logic, we generated simulated behavioral categorization responses and response confusion matrices based on our three representation models. Note that categorization is a complex process consisting of multiple components (e.g., acoustic analysis, perception, discrimination, and decision-making processes), whereas the three models we proposed are only about the representations (may overlap with the perception and parts of the discrimination components). Thus, we consider this simulation to be experimental and exploratory instead of a confirmational demonstration that mimics auditory categorization.

We found that each of the simulated behavioral RDMs was significantly correlated with their source model RDMs in the mean performance levels of 0.8 and 0.95 (*P*s < 0.001, non-parametric test; also refer to the RSA model fit distributions below). No significant RSA model fit was found for the random responders (mACC = 0.5).

To further examine whether the simulated response RDMs derived from the bound-based distance and center-based PS models contain *item-level* distance or PS information in addition to the *category-level* confusions, we re-conducted the same RSA while controlling for the variances of the binary category model with a partial correlation approach. We found that the simulated response RDMs still significantly correlated with their sources model RDMs even controlled for the binary category RDM (bound-based distance model: mACC = 0.95, mean rho = 0.163, *P* < 0.001; mACC = 0.80, mean rho = 0.084, *P* < 0.001; mACC = 0.50, mean rho = 0, *P* > 0.2; center-based PS model: mACC = 0.95, mean rho = 0.097, *P* < 0.001; mACC = 0.80, mean rho = 0.059, *P* < 0.001; mACC = 0.50, mean rho = 0, *P* > 0.2). We can also see that the binary model explains a large portion of the variances. However, even when controlled for the binary model, the distance and PS information can explain the item-level confusion and responses.

**Supplementary Figures**


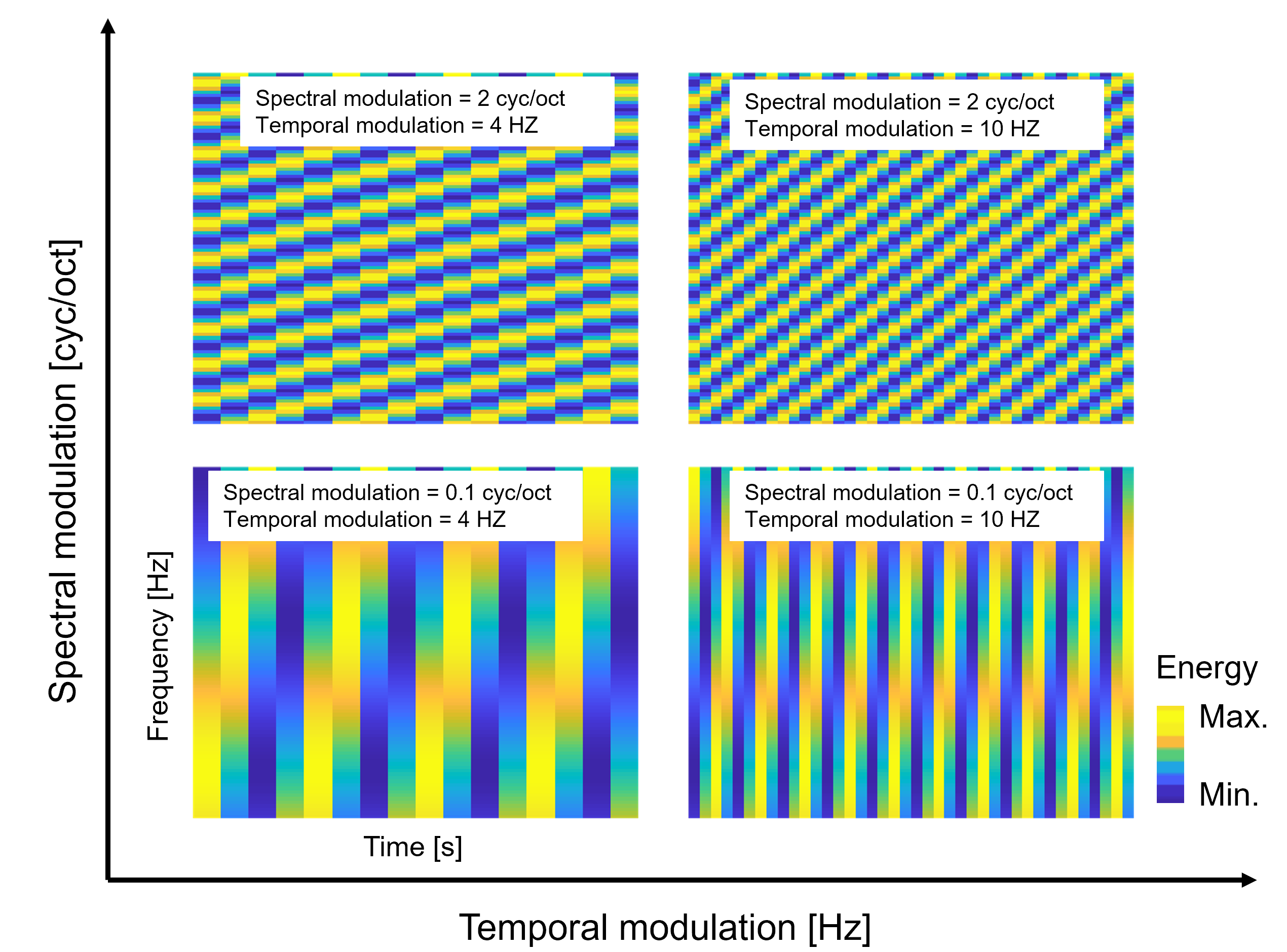


**Figure S1**. Graphical illustration of spectral and temporal modulations of the stimuli used in the experiments. Note that these graphs are not the spectrograms of sounds (see the spectrograms in Fig. 1). They are the visual representations of the auditory modulation parameters (i.e., temporal and spectral modulations) for readers to understand the acoustic differences between sounds from different categories.


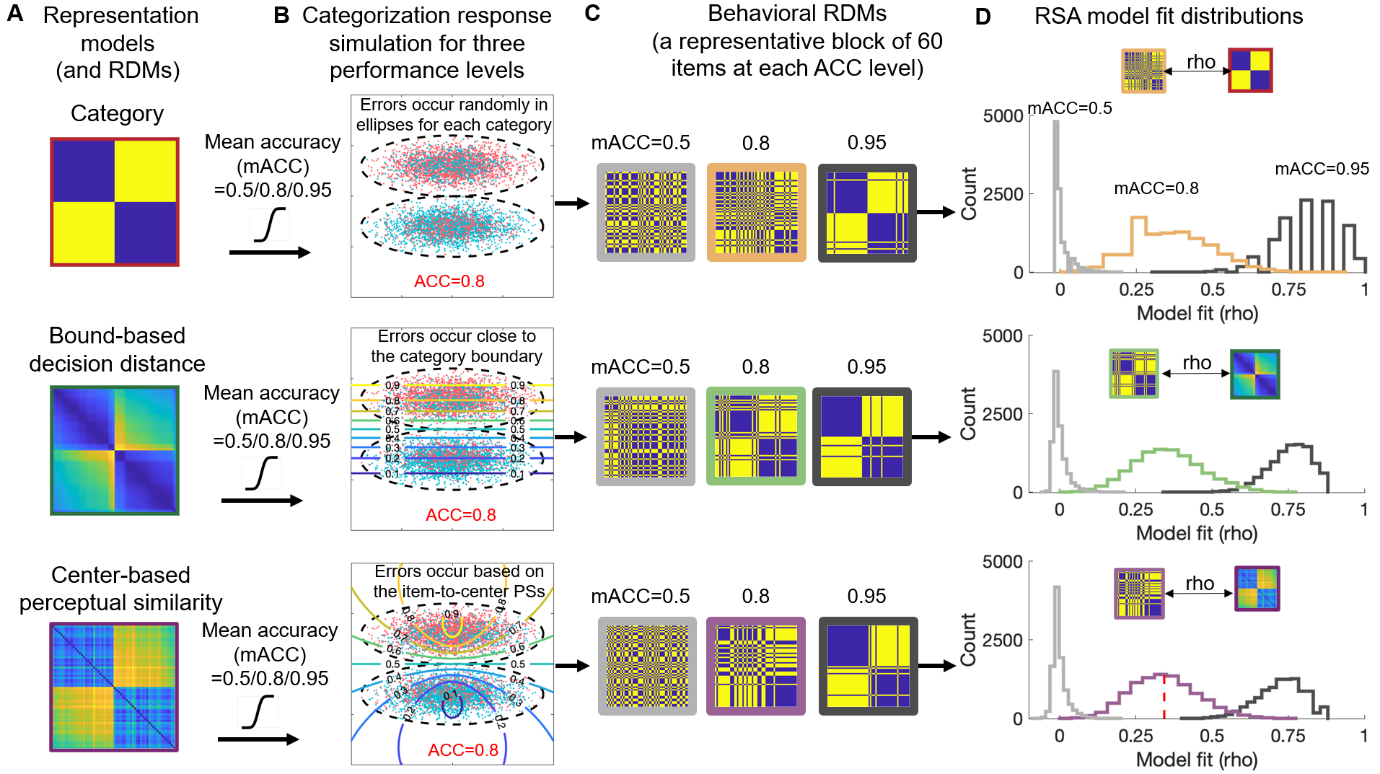


**Figure S2**. Categorization simulation based on the three models and RSA modeling with simulated data. **A**, three pre-defined representation models and their corresponding RDMs. **B**, categorization response simulations with the three models with performance levels of mean accuracy = 0.5, 0.8, and 0.95. The colored lines or curves in the scatter plot (the 2^nd^ and 3^rd^ rows) are response probability distributions of Category 1. Higher numbers indicate more likely to respond to category 1. These lines or curves are calculated based on the item-level item-to-bound distance and item-to-center PS for the bound-based distance and center-based PS models, respectively. **C**, behavioral RDMs computed from the simulated responses for the three models. **D**, RSA modeling results. The simulated response RDMs were correlated with their source model RDMs at different accuracy levels.


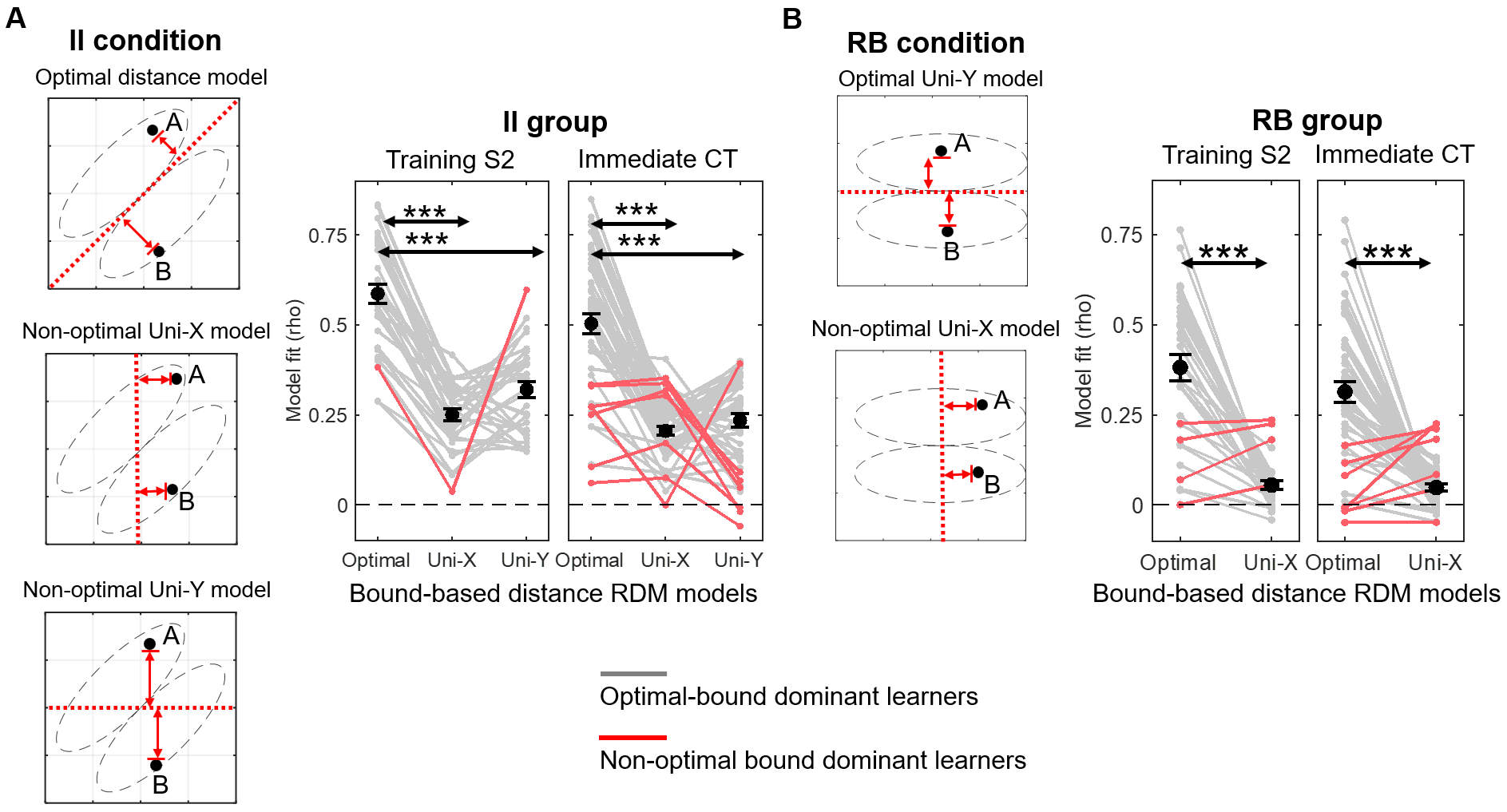


**Figure S3.** Control RSA analyses with new hypothetical RDMs to classify individual learners into optimal-bound dominant and non-optimal-bound dominant learners. **A**, three bound-based distance models for II condition. **B**, optimal and non-optimal distance models for RB conditions. Red lines, RSA model fits are higher with the non-optimal models compared to the optimal models.


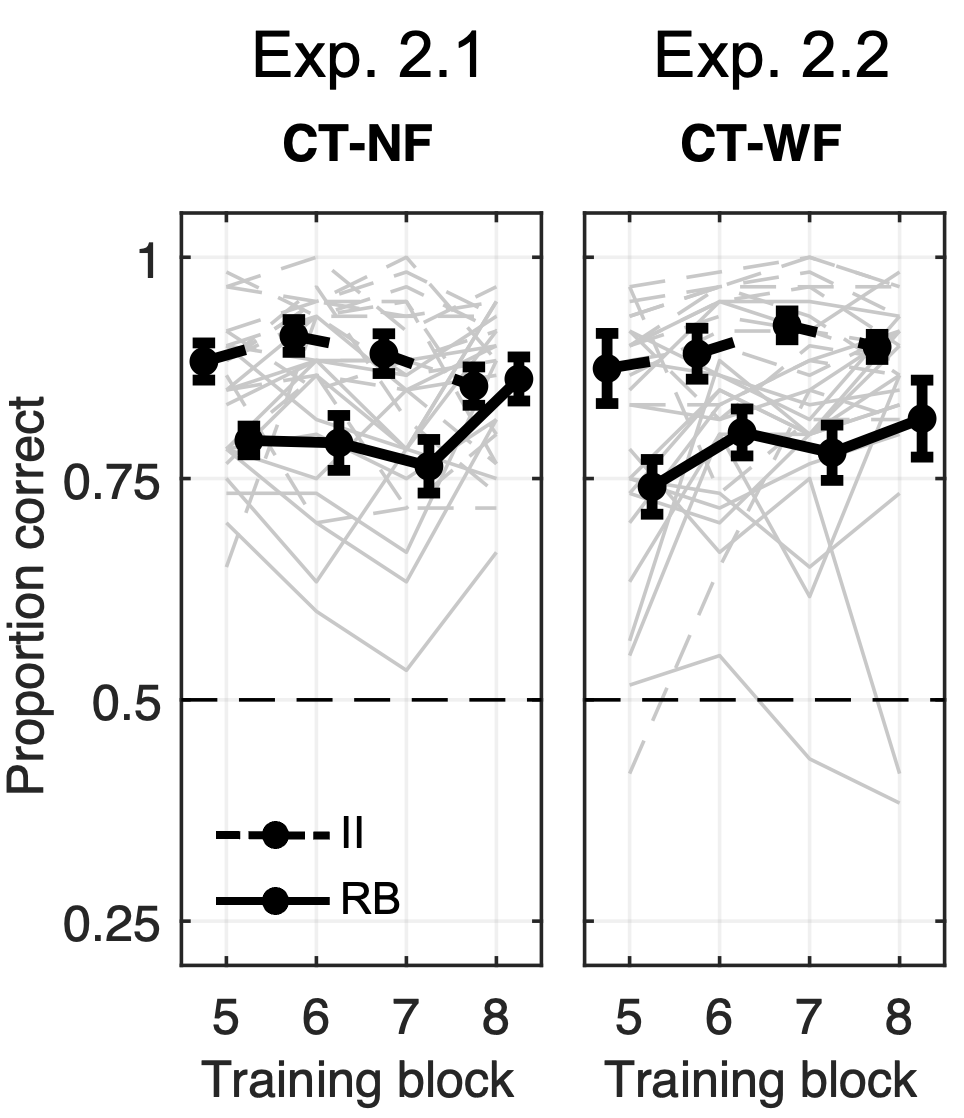


**Figure S4.** Block-by-block categorization performances (proportion correct) during training session 2 for Experiment 2.1 (CT-NF) and 2.2 (CT-WF). CT-NF = categorization training no feedback; CT-WF = categorization training with feedback.


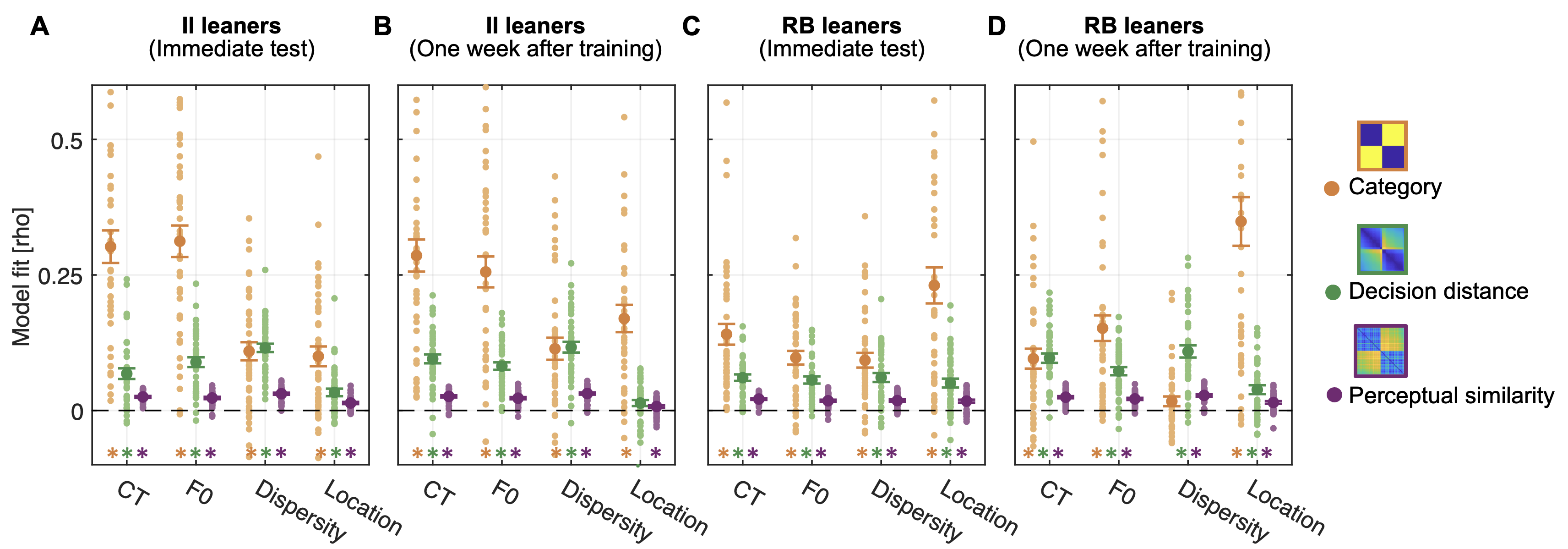


**Figure S5**. Representational similarity modeling of categorization response patterns for each generalization test with the three pre-defined models. Unique contributions of each model to the behavioral responses were shown while controlling for the variance of other models. Data were collapsed across three experiments. *, corrected *p* < 0.05 above chance.


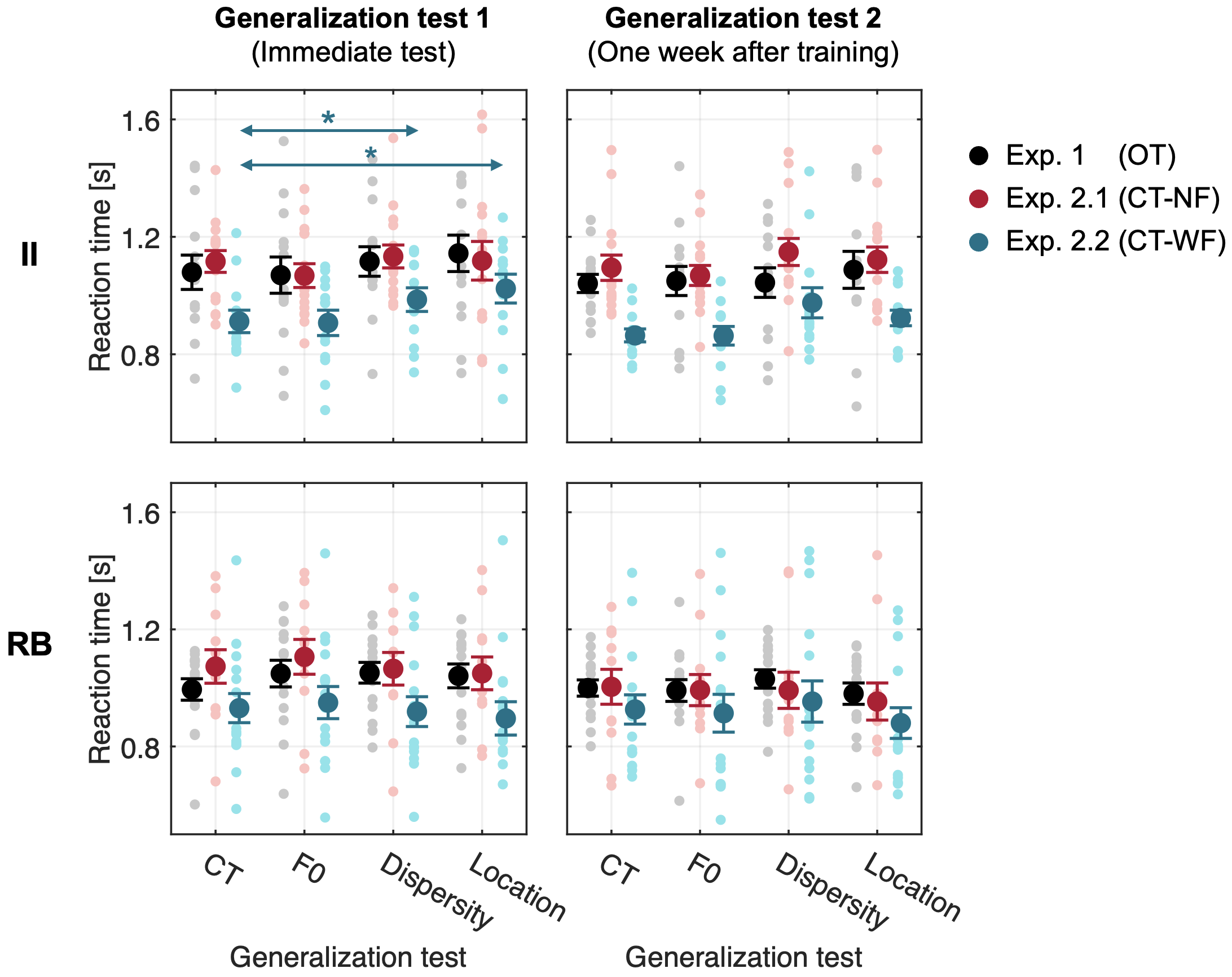


**Figure S6.** Learners’ categorization response time for each learner group, generalization test, and experiment across two testing time points. *, *p* < 0.05.
